# Supplementary material for: How can we strengthen mental health services in Swedish youth clinics? A health policy and systems study protocol
Source: BMJ Open. 2021 Oct 22;11(10):e048922. doi: 10.1136/bmjopen-2021-048922 (PMC8543652; doi:10.1136/bmjopen-2021-048922)
Supplement: Author's manuscript [file bmjopen-2021-048922.draft_revisions.pdf]

BMJ Open is committed to open peer review. As part of this commitment we make the peer review history of every article we publish publicly available.

When an article is published we post the peer reviewers' comments and the authors' responses online. We also post the versions of the paper that were used during peer review. These are the versions that the peer review comments apply to.

The versions of the paper that follow are the versions that were submitted during the peer review process. They are not the versions of record or the final published versions. They should not be cited or distributed as the published version of this manuscript.

BMJ Open is an open access journal and the full, final, typeset and author-corrected version of record of the manuscript is available on our site with no access controls, subscription charges or pay-per-view fees (http://bmjopen.bmj.com).

If you have any questions on BMJ Open's open peer review process please email info.bmjopen@bmj.com

BMJ Open

How can we strengthen youth mental health services in Swedish youth clinics? A health policy and systems study protocol

Journal:	*BMJ Open*
Manuscript ID	bmjopen-2021-048922
Article Type:	Protocol
Date Submitted by the Author:	13-Jan-2021
Complete List of Authors:	Richter Sundberg, Linda; Umea University, Department of Epidemiology and Global Health<br>Christiansson, Monica ; Umea University, Department of Nursing<br>Wiklund, Maria; Umea University, Department of Community Medicine and Rehabilitation<br>Hurtig, Anna-Karin ; Umea University, Department of Epidemiology and Global Health<br>Goicolea, I; Umea University, Department of Epidemiology and Global Health
Keywords:	MENTAL HEALTH, Organisation of health services < HEALTH SERVICES ADMINISTRATION & MANAGEMENT, Child & adolescent psychiatry < PSYCHIATRY

SCHOLARONE™
Manuscripts

I, the Submitting Author has the right to grant and does grant on behalf of all authors of the Work (as defined in the below author licence), an exclusive licence and/or a non-exclusive licence for contributions from authors who are: i) UK Crown employees; ii) where BMJ has agreed a CC-BY licence shall apply, and/or iii) in accordance with the terms applicable for US Federal Government officers or employees acting as part of their official duties; on a worldwide, perpetual, irrevocable, royalty-free basis to BMJ Publishing Group Ltd ("BMJ") its licensees and where the relevant Journal is co-owned by BMJ to the co-owners of the Journal, to publish the Work in this journal and any other BMJ products and to exploit all rights, as set out in our licence.

The Submitting Author accepts and understands that any supply made under these terms is made by BMJ to the Submitting Author unless you are acting as an employee on behalf of your employer or a postgraduate student of an affiliated institution which is paying any applicable article publishing charge ("APC") for Open Access articles. Where the Submitting Author wishes to make the Work available on an Open Access basis (and intends to pay the relevant APC), the terms of reuse of such Open Access shall be governed by a Creative Commons licence – details of these licences and which Creative Commons licence will apply to this Work are set out in our licence referred to above.

Other than as permitted in any relevant BMJ Author's Self Archiving Policies, I confirm this Work has not been accepted for publication elsewhere, is not being considered for publication elsewhere and does not duplicate material already published. I confirm all authors consent to publication of this Work and authorise the granting of this licence.

For peer review only

How can we strengthen youth mental health services in Swedish youth clinics? A health policy and systems study protocol

Linda Richter-Sundberg[1,*], Monica Christianson[2], Maria Wiklund[3], Anna-Karin Hurtig[1], Isabel Goicolea[1]

[1]Department of Epidemiology and Global Health Umeå University, Umeå, Sweden

[2]Department of Nursing, Umeå University, Umeå, Sweden

[3]Unit of Physiotherapy, Department of Community Medicine and Rehabilitation, Umeå University, Umeå, Sweden

*Correspondence: linda.sundberg@umu.se

Word count 3963

For peer review only

Abstract

Introduction

Strengthening first-line mental health care services for youth remains a priority for the Swedish government. The government is currently investigating how different sectors involved can be strengthened, but evidence is scarce. Youth clinics play a key role in these discussions, being one of the most trusted services for youth. However, analysis of organizational functions and coordination with other services is important to strengthen youth-clinics role in first-line mental health care. This study investigates these challenges and aims to analyze the integration of mental health care within youth clinics to identify strategies to strengthen first-line mental health care for youth in Sweden.

Methods and analysis This study adopts a health policy and systems approach. In the first phase, a formative realist evaluation is conducted to ascertain what works in terms of integrating mental health care services within youth clinics, for what type of youth subpopulations, and under what circumstances. National-level stakeholders will be interviewed to elicit the program theory that explains how the intervention is supposed to work. The program theory will then be tested in 3-5 cases. Quantitative and qualitative information will be gathered, including visual methodologies and questionnaires. The second phase includes a concept mapping study, engaging stakeholders and young people to build consensus on strategies to strengthen the integration of mental health care into youth clinics.

Ethics and dissemination The Swedish Ethical Review Authority has approved the study (2019-02910, 2020-04720). The results will be published in open-access peer-reviewed journals and presented at scientific conferences.

For peer review only

For peer review only - http://bmjopen.bmj.com/site/about/guidelines.xhtml

Keywords: Implementation, Mental health, Youth health systems, Primary health care, Integration, Youth clinic

Article Summary

Strengths and limitations

- The study addresses an urgent problem in health systems globally – the barriers for youth access to high quality mental health services.
- The study will generate knowledge to strengthen first-line mental health care for youth in Sweden and in similar settings where differentiated services for youth that integrate mental health care are being developed.
- This study will add the team and organizational levels to the individual level, which might challenge the method of realist evaluation to some degree.
- A challenge of the study is to ensure meaningful participation of young people, especially in the concept mapping step of the study

Introduction

In Sweden, the prevalence of mental health problems among young people has increased about 70% during the period 2006–2016, the highest increase among the Nordic countries. Depression, anxiety, and substance abuse have shown the largest increase [1, 2], and suicide rates have not decreased in younger age groups as they have in the overall population [2, 3]. This development is one of the major challenges for Swedish health policies, but are not unique to Sweden; similar trends are seen in other countries [4].

For peer review only - http://bmjopen.bmj.com/site/about/guidelines.xhtml

More than 75% of mental health problems have onset during childhood or adolescence, therefore this is a period of opportunity for health systems to promote youth mental health and respond to youth's mental health problems in coordination with other actors and sectors [4, 5]. However, international research shows that access to mental health services for youths is often hindered by factors such as long waiting times, youth concerns about confidentiality, and lack of trust in professionals [5, 6]. The transfer between youth mental health services (e.g., from child mental health services to adult mental health services) is experienced by young people as dramatic and with poor coordination [7].

To enhance accessibility to mental health care services for youth, reduce waiting times and facilitate coordination, the Swedish health system since 2009 has been developing and gradually adopted a strategy to integrate youth mental health services within primary care services, the so-called First-Line Mental Health Services (FLMHS) [8]. This approach has been used in several other countries [9,10]. Research has shown that the integration of mental health services within primary care improves the management of common mental health problems among adults [11, 12] and children [13]. Youth between 15 and 25 years of age found it easier to access mental health services when delivered through less stigmatized, more visible and familiar primary care services [14]. Reforms of youth mental health services have been implemented in many countries, but surprisingly, there is still limited research analyzing integrated care models such as the FLMHS [14–16].

To improve youth access to mental health services in Sweden, in 2009 the Ministry of Health gave the Swedish Association of Local Authorities and Regions a new assignment to further develop and implement FLMHS for children and youth [17]. It was launched as a way to address three main challenges: (1) inequities in the mental health and social services provided to children and youth, (2) unclear division of responsibilities within and between health and social services, and (3) a need to develop versatile health system solutions

For peer review only - http://bmjopen.bmj.com/site/about/guidelines.xhtml

reflecting the diverse conditions prevailing in the different groups of the population and parts of the country. The establishment of FLMHS was in addition expected to relieve the high (unfulfilled) demand for specialized child mental health services and strengthen the collaboration between diverse public services and sectors [17, 18]. Currently, the sectors that are most frequently involved in FLMHS are primary health care centers, school health services and youth clinics [19].

In Sweden, more than 250 youth clinics (YCs) have offered youth-centered health services for young people since the 1970–1980s. This national network of YCs offers an important opportunity to integrate mental health services within services that report a high level of trust by young people [20].

In this paper, we present the protocol for a health policy and system study to analyze the policy efforts in Sweden to strengthen FLMHS for youth, focusing on YCs and adopting the conceptual framework for investigating the integration of health interventions into health systems according to Atun et al. [21]. The research questions we aim to answer with this study are as follows: (1) How and under what circumstances are YCs part of the FLMHS for youth in the Swedish health system? (2) How can the integration of mental health services into YCs in the Swedish health system be strengthened?

Conceptual framework

The approach to mental health systems used in this study is inspired by complexity theory [22], with a specific focus on the integration of targeted health interventions within health systems [21]. Instead of portraying the different hierarchical levels and actors in the mental health system [17], complexity theory portrays them as interconnected and centered in the user of the services (Fig. 1). In complex adaptive systems, individuals, services, and

For peer review only - http://bmjopen.bmj.com/site/about/guidelines.xhtml

organizations are active agents that interact with each other, establishing dynamic interconnections [22].

<Figure 1 The Model for Mental Health Care Delivery as a Complex Adaptive System inspired by Ellis et al [22].>

This study will explore the intentions and strategies among national stakeholders and policy makers (e.g., authorities, politicians) in terms of integrating YCs in FLMHS and if and how YCs coordinate with other health actors in the FLMHS for youth, with specialized mental health services and with social services. The organization of this system is influenced (and influences) both policies and research (Fig. 1).

Inspired by Atun et al. [21], we conceive integration as the process, pattern, and extent of adoption and eventual assimilation of new strategies into the health system (in this case, mental health services into YCs) that are embedded within FLMHS for youth.

In Figure 2, we have adapted Atun et al.'s framework to the specific intervention being analyzed in this study. Atun et al.'s conceptual framework provides a systematic approach to researching integration by focusing on 5 domains that influence the adoption and diffusion of health interventions.

<Figure 2 Conceptual Framework to Analyze Integration of Mental Health Care for Youth within Swedish Youth Clinics>

For peer review only - http://bmjopen.bmj.com/site/about/guidelines.xhtml
For peer review only

The first domain relates to the nature of the problem being addressed, i.e., the understanding regarding the urgency and severity of the problem influences the speed of adoption [21]. In this protocol, we conceptualize the problem as the weaknesses of the health system to adequately respond to the increasing mental health needs of young people.

The second domain refers to the characteristics of the intervention. Here, Atun et al. point out that interventions that for example are perceived as having a relative advantage, are less complex and risky, are more easily integrated. In this protocol, the intervention to address this problem is the integration of mental health services within the existing network of Swedish YCs.

The third domain refers to the adoption system, namely the key actors and institutions involved in the integration [21]. Each stakeholder has differing perceptions of the benefits, risks, and legitimacy of the intervention, and the different interests at a stake have to be explored. The adoption system in this study focuses on the YC care providers, the managers, and the young users.

The fourth domain refers to the characteristics of the health system [21]. With regard to the characteristics of the health system, we are interested in the larger mental health system (i.e., national governance, primary and specialized mental health and social services), but with special focus on the sub-system of FLMHS for youth. The focus is on analyzing the collaboration between the services responsible for FLMHS for youth and how the different ways of organizing them and their approaches toward young people influence both the integration of mental health services within YCs and the collaborative dynamics with the other services.

The fifth domain refers to the broader socio-cultural context, where critical events, prevailing political economy, and socio-cultural norms also affect integration [21]. The broader context in the study protocol refers to how the current political economic situation

For peer review only - http://bmjopen.bmj.com/site/about/guidelines.xhtml
For peer review only

and the socio-cultural characteristics of Swedish society, influence the integration of mental health services for youth within YCs.

Material and methods

Study design

To address our two main research questions, two methodological approaches will be combined in a sequential way: (1) a realist evaluation [25] and (2) concept mapping [26]. The study design will follow an iterative analytical process in which the conduct of the concept mapping will draw on the findings from the realist evaluation. Qualitative and quantitative data collection and analysis will be combined to best capture the complexity of the phenomenon under investigation. The integration of qualitative and quantitative approaches enables researchers to explore phenomena from different perspectives and provides an enriched understanding [27]. The data collection started in November 2019 and will be finalized in November 2022. The main research questions and the methodologies applied are summarized in Table 1.

For peer review only

Table 1: The research process

Research question in focus	Methodological approach	Steps	Methods
How and under what circumstances are YCs part of the FLMHS for youth in the Swedish health care system?	Realist evaluation	Eliciting the program theory	Literature review
			Documentary review
			Interviews with stakeholders
		Testing the program theory in selected cases	Analytical multiple case study
			Questionnaires for mental health care consultations
		Refining the program theory	Interviews with professionals from YCs
			Interviews with professionals from other services involved in FLMHS for youth
			Interviews with professionals at specialized services
			Photo-elicitation with young people

For peer review only - http://bmjopen.bmj.com/site/about/guidelines.xhtml

How can the integration of mental health care services into YCs in the Swedish health system be strengthened?	Concept mapping	Presenting the findings and engaging professionals and young people	Workshop and photo exhibition with (1) professionals working in YCs, (2) professionals in other FLMHS services for youth and in managerial positions, and (3) young people and youth organizations
		Developing the concept maps	Brainstorming, pile sorting and rating, and map development using Concept System-CS Global Max
		Bringing everything together and proposing actions	Final workshop

Patient and public involvement

The research questions in this study were informed by the experiences and preferences of youth, through our previous studies on youth views on YCs. Further the study adopts a participatory approach which has implications for the design, conduct of the study and also the choice of outcome measures.

All phases of the study will be presented and discussed with stakeholders at youth clinics (both managers and professionals) and other arenas (i.e. youth organizations) before and during data collection. Dissemination is a core component of the second phase, stakeholders (including young people) will be invited to workshops to discuss preliminary results and engage in a concept mapping to collectively develop a set of strategies to improve the integration of mental health services within YCs. One of the main outcomes in this study is to reach consensus on the strategies to strengthen the integration of youth clinics in the FLMHS.

Study setting

The responsibility for providing health services in the Swedish health care system is shared between the national government, the regions, and the municipalities. The Swedish Health Care Act gives a commitment to provide "good health and care on equal terms for the entire population" [23]. However, evaluations have shown that evidence-based health services are

For peer review only

inaccessible to children and youth with mental health problems to an extent that diverges from the Health Care Act [24].

Data collection and analysis phase I - Formative realist evaluation

To answer the first research question, a formative realist evaluation of the integration of mental health services within YCs will be conducted. Realist evaluation is well suited to analysis of complex interventions aiming to ascertain whether the intervention works but also why, for whom, and under what circumstances [25,28]. In realist evaluation, theories are at the middle-range level, connecting the intervention with contextual factors, with the mechanisms that the intervention triggers within the target populations, and with the outcomes achieved. The aim is to identify patterns of intervention-context-mechanism-outcomes that can explain how, why, and under what circumstances a specific intervention works.

Pawson [25] describes cycles of realist evaluation, starting from eliciting the program theory behind the implementation of a particular intervention, followed by putting this theory under test in specific cases and coming up with a refined program theory. The entire process should be conducted in close dialogue with stakeholders involved in the development and implementation of the intervention, because the aim is to come up with a revised and improved intervention [25].

Realist evaluation can be applied in a summative way (once an intervention has finalized) or in a formative way (while an intervention is being implemented). Integrated care programs for young people's mental health need to adapt and learn continuously, therefore we view our realist evaluation through the lens of formative evaluations, aiming for continuous quality improvement. The realist evaluation will cover the following steps: (1) developing the program theory, (2) testing the program theory in selected cases, (3) refining the program theory.

For peer review only - http://bmjopen.bmj.com/site/about/guidelines.xhtml

Developing the program theory

The realist evaluation will start developing the program theory through a review of the literature, policy documents, and interviews with stakeholders. The scientific literature, policies, and other relevant documents related to the intervention will be reviewed, looking for key concepts, ideas, and theories that can help to explain how the integration of mental health services for youth within the FLMHS is supposed to work.

In addition, we will interview national- and regional-level stakeholders involved in developing the intervention (i.e., the Swedish Association of Local Authorities and Regions, The Swedish Association of Youth Clinics). The interviews will aim to make the program theory behind the intervention explicit, namely the integration of mental health services within YCs, and to analyze how the increasing problems of youth mental health and the planned solutions/responses are understood. The five domains of Atun et al.'s framework [21] in relation to the three key concepts of realist evaluation (context, mechanisms, outcomes) will be the main topics explored. The interviews will be audio-recoded, transcribed, and analyzed using abductive thematic analysis following Clarke & Braun [29], moving back and forward between our conceptual framework and the data. The program theory that develops will explain how the intervention is planned to work. Following the ideas of FLMHS [17], the outcomes will mainly focus on whether the YCs (1) are accessible, (2) allow early detection, (3) have broad expertise of good quality, (4) have good reporting and monitoring systems, and (5) have good collaboration with other services [17].

Testing the program theory in selected cases

In the next phase, the program theory will be put to the test in 3–5 specific cases: YCs and the FLMHS in which they are embedded, including the surrounding referral services. To capture diverse contextual circumstances, we will choose clinics in different municipalities,

For peer review only - http://bmjopen.bmj.com/site/about/guidelines.xhtml

located in different regions, and embedded in different models of FLMHS for youth (as defined by the Swedish Association of Local Authorities and Regions [15]).

From each case, quantitative information will be gathered on the extent and characteristics of mental health consultations. For this study, we will distribute the questionnaire to all the professionals working at the YC in the 3–5 cases, including midwives, psychologists, social workers, medical doctors, and other staff who are frequently approached by young people to discuss mental health issues. The questionnaire will be applied over 6 months in each of the clinics participating as cases. Analysis of the quantitative data will offer answers whether the services (1) are accessible, (2) allow early detection, and (3) offer good quality services [18].

Qualitative information from each case will include interviews (~5–10 per case) with (1) professionals working in the YC, (2) professionals working in other FLMHS for youth (e.g., student health, health centers, social services), and (3) professionals working in specialized mental health services. Because the way different municipalities are implementing FLMHS for youth and the services with responsibility for this sub-system vary, the institutions represented might vary from case to case. Professionals working in the YCs will include the head of the clinic and a variety of professionals, including professions more focused on mental health (e.g., counselor, psychologist) and others (e.g., midwives). Interviews at this stage will focus on health professionals' experiences of mental health consultations and if and how mental health services have been integrated and possibly sustained within the clinic's practice, coordination within the clinic and with other health care resources (both first-line and specialized) and social resources. Professionals working in FLMHS for youth will include those working in, for example, primary health care centers and school health services. Professionals working in specialized mental health services will involve child mental health services and adult mental health services. Interviews with staff in specialized mental health

For peer review only

For peer review only - http://bmjopen.bmj.com/site/about/guidelines.xhtml

services will focus on how they perceive the role of YCs in FLMHS for youth, and their collaboration with YCs and with other services.

To capture the views and experiences of young people (aged 16–25 years), we will engage in an auto-driven photo-elicitation interview study with young people to gather their perspectives [29, 30]. Photo-elicitation is a research method that incorporates images in the interview process to elicit participants' subjective explanations. Photo-elicitation has gained recognition as a way to better capture young people's attention, ease rapport, and balance power differentials.

From each case, 6–8 youths aged 16–29 years will be recruited. Participants will include youth with experience using YC mental health services and those who do not have that experience. For the latter, we will recruit young people from high schools and universities. First, each participant will be approached and receive information about the study, offered a digital camera for 2 months and asked to take pictures to depict young people experiences and/or expectations of mental health and/or mental health services in the area where they live. At a second meeting, the participant will select 3 of the pictures taken as the basis for an interview, where topics around youth mental health, access to services, and experiences of them (with special focus on YCs) will be discussed.

All the interviews will be audio-recorded and transcribed. Detailed descriptions of the cases will be developed and further analyzed using an abductive thematic analysis approach, guided by the framework presented above (Fig. 2). This analysis will inform about intervention implementation, contextual factors, potential mechanisms, and outcomes.

Refining the program theory

The initial program theory will be refined based on the findings in the theory testing to specify a middle-range theory that describes what works in terms of integrating mental health

For peer review only - http://bmjopen.bmj.com/site/about/guidelines.xhtml

services for young people within YCs, for what type of youth subpopulations, and under what contextual circumstances [25, 28].

Data collection and analysis phase II – A concept mapping study

Building upon the knowledge gained during the previous phase, we will engage in a participatory process in which professionals and young people will be invited to take part in workshops followed by a concept mapping study [26] to develop strategies to strengthen the integration of mental health services within YCs. The concept mapping entails three steps: presenting findings and engaging professionals and young people in participatory workshops, developing the concept maps, and bringing everything together and proposing actions.

Presenting findings and engaging professionals and young people in participatory workshops

To present the preliminary results of the study, workshops will be arranged with 3 different types of stakeholders: (1) professionals working in YCs, (2) professionals in other FLMHS for youth and in managerial positions, and (3) young people and youth organizations. Youth will be recruited to the workshop using three strategies: first, the youth who were involved in the previous interview study in the project will be asked to participate in the workshop; second, YCs involved in the cases will be asked to invite youth through advertising in waiting rooms/reception areas; and third, youth delegations will be asked to nominate youth to participate in the workshop.

Developing the concept maps

Using the findings from the participatory workshops as a basis for reflection, participants will be asked to participate in a concept mapping study to collectively develop a set of strategies to improve the integration of mental health services within YCs.

For peer review only - http://bmjopen.bmj.com/site/about/guidelines.xhtml

Concept mapping enables groups of actors to visualize their ideas about an issue of mutual interest and to develop common frameworks through a structured, participatory process. Qualitative and quantitative data are generated and integrated by participants through sequential steps, beginning with the generation of ideas (brainstorming), structuring of ideas through sorting and rating, development of conceptual maps based on multivariate statistical methods, and collective interpretation of the maps [26].

The research team will develop a focus question to guide the brainstorming activity and with clear instructions to all workshop participants and other relevant stakeholders and young people. Once the refined list of actions is developed, it will be sent again to the participants for sorting and rating. Three different sets of maps (one for each group) will be developed. The data will be analyzed using concept mapping techniques, which facilitate visualization of thematic clusters and identification of areas of consensus for action. The Concept System-CS Global Max software will be used throughout the process.

Bringing everything together and proposing actions

Finally, the concept maps and all the qualitative and quantitative results will be synthesized to answer our main research question: "How can the integration of mental health services into YCs in the Swedish health system be strengthened?" Building on the concept maps and synthesized results, a final workshop will be held with young people and relevant stakeholders to discuss the maps and come up with a set of elaborated strategies.

Ethics and dissemination

This study has received approval from the Swedish Ethical Review Agency (Dnr 2019-2910, 2020-04720) and complies with the Helsinki Declaration. All participants will be informed that participation in the study is voluntary. Individuals will be provided with information in

For peer review only

verbal and written format, and offered the opportunity to ask any questions. Written informed consent will be obtained from all participants.

Results will be published in open-access peer-reviewed journals and presented at scientific conferences, and as described before, preliminary findings will be discussed with stakeholders during the concept mapping.

Discussion

This paper presents a study protocol using a multiple case-study design to understand the integration of mental health services within YCs with a view to strengthening FLMHS for youth in Sweden and to develop strategies for strengthening this process.

This study is timely as it goes hand in hand in informing policy efforts to integrate mental health services within YCs and YCs within the FLMHS for youth in Sweden. Atun et al.'s [21] framework has been used to analyze the integration of programs on communicable diseases, cardiovascular disease, and sexual and reproductive health interventions, but, to our knowledge, it has never been used to analyze the integration of mental health services in primary care. The methodological approach, involving realist evaluation and concept mapping, combines quantitative and qualitative methods sequentially in a way that fits the research questions and the health systems approach to complexity, and is innovative in the field of public health.

The study combines two methodological approaches: realist evaluation [25] and concept mapping [26]. Unlike randomized controlled trials, realist evaluation does not aim to control contextual factors but rather to explore how outcomes of an intervention interact with the context in which it is implemented. Realist evaluation is a methodology that rests on critical

For peer review only - http://bmjopen.bmj.com/site/about/guidelines.xhtml

realism, and it mainly focuses on individual mechanisms in understanding an intervention. In this study, we do not focus only at the individual level but also the team and organizational levels. This challenges the method to some degree, but we have good experiences of using the method in this broader sense, for example, in the context of primary health care services [31].

Concept mapping is a mixed participatory method for involving stakeholders and youth in a structured conceptualization process. It has been developed to collect and integrate input from multiple sources and stakeholder with various demand, expertise or interest [32]. One challenge in using concept mapping is that the methodology utilizes group processes, for example, in brainstorming activities. Interaction within groups and the group processes are affected by entities such as power and status [33]. As participants are diverse in terms of age, education, and gender, we see a potential risk that participants not will have equal possibilities to contribute in the concept mapping. To balance participant contributions in group-oriented tasks, we will have this challenge in mind as we put the groups together. The moderator of the session will also be instructed to facilitate the participation of all group members. By taking these measures, we hope to balance the potential negative impact of the group processes to some degree.

The results of the study are expected to contribute new knowledge concerning the process of integration of YC in the FLMHS for youth but also to lead to recommendations for policy makers and decision makers within the field of youth mental health services.

Authors' contributions

IG designed the research proposal, drafted the application, and led the discussion in the group as principal investigator of the research proposal. LRS led the development of the first

For peer review only - http://bmjopen.bmj.com/site/about/guidelines.xhtml

draft of the manuscript based on the research proposal and gathered comments from all team members to revise successive drafts and develop and submit the final draft of the manuscript to the journal. IG, MC, MW, and AKH are part of the research team that supported and developed the protocol. They contributed with ideas and discussed the protocol that was submitted and granted funding from FORTE. They read and critically commented on and revised successive drafts of the manuscript and approved the final version before submission to the journal.

Funding statement

This work was supported by the Swedish Research Council for Working Life and Living Conditions (FORTE) grant number 2018-00364.

Competing interests

The authors declare that they have no competing interests.

References

1. Bremberg S. Mental health problems are rising more in Swedish adolescents than in other Nordic countries and the Netherlands. Acta Paediatr. 2015;104(10):997–1004.

2. Public Health Agency (Folkhälsomyndigheten). Skolbarns hälsovanor i Sverige 2013/2014. Stockholm: Folkhälsomyndigheten; 2014.

3. National Board of Health and Welfare (Socialstyrelsen). Utvecklingen av psykisk ohälsa bland barn och unga vuxna till och med 2016. Stockholm: Socialstyrelsen; 2017.

For peer review only

4. Weinberger A, Gbedemah M, Martinez A, Nash D, Galea, S, Goodwin R. Trends in depression prevalence in the USA from 2005 to 2015: widening disparities in vulnerable groups. Psychol Med. 2018;48(8):1308–15.

5. Patel V, Flisher AJ, Hetrick S, McGorry P. Mental health of young people: a global public-health challenge. Lancet. 2007;369(9569):1302–13.

6. Malla A, Iyer S, McGorry P, Cannon M, Coughlan H, Singh S, et al. From early intervention in psychosis to youth mental health reform: a review of the evolution and transformation of mental health services for young people. Soc Psychiatry Psychiatr Epidemiol. 2016;51(3):319–26.

7. Gulliver A, Griffiths KM, Christensen H. Perceived barriers and facilitators to mental health help-seeking in young people: a systematic review. BMC Psychiatry 2010;10(1):113.

8. Broad KL, Sandhu VK, Sunderji N, Charach A. Youth experiences of transition from child mental health services to adult mental health services: a qualitative thematic synthesis. BMC Psychiatry. 2017;17(1):380–11.

9. Swedish Association of Local Authorities and Regions (Sveriges Kommuner och Regioner). Uppdrag Psykisk Hälsa. Första linjen för barn och ungas psykiska hälsa. Stockholm: SALAR; 2016.

10. World Health Organization. Integrating mental health into primary care: a global perspective. Geneva: World Health Organization; 2008.

11. Druss BG, Goldman HH. Integrating Health and Mental Health Services: A Past and Future History. American Journal of Psychiatry. 2018 Dec 1;175(12):1199–204.

For peer review only

For peer review only - http://bmjopen.bmj.com/site/about/guidelines.xhtml

12. Kates N, Arroll B, Currie E, Hanlon C, Gask L, Klasen H, et al. Improving collaboration between primary care and mental health services. The World Journal of Biological Psychiatry. 2019 Nov 26;20(10):748–65.

13. Campo JV, Geist R, Kolko DJ. Integration of pediatric behavioral health services in primary care: improving access and outcomes with collaborative care. Can J Psychiatry. 2018;63(7):432–38.

14. Hetrick SE, Bailey AP, Smith KE, Malla A, Mathias S, Singh SP, et al. Integrated (one-stop shop) youth health care: best available evidence and future directions. Med J Aust 2017;207(S10):S5–18.

15. Kinchin I, Tsey K, Heyeres M, et al. Systematic review of youth mental health service integration research. Aust J Prim Health. 2016;22(4):304–15.

16. Richardson LP, McCarty CA, Radovic A, Suleiman AB. Research in the integration of behavioral health for adolescents and young adults in primary care settings: a systematic review. J Adolescent Health. 2016;60(3):261–9.

17. Swedish Association of Local Authorities and Regions (Sveriges Kommuner och Regioner). Uppdrag Psykisk Hälsa. Första linjen för barn och ungas psykiska hälsa. Stockholm: SALAR; 2016.

18. Swedish Association of Local Authorities and Regions (Sveriges Kommuner och Regioner). Psykisk hälsa på ungdomsmottagningen – en kartläggning av Sveriges ungdomsmottagningar. Stockholm: SALAR; 2017.

19. Swedish Association of Local Authorities and Regions (Sveriges Kommuner och Regioner). Uppdrag Psykisk Hälsa. Första linje boken. Stockholm: SALAR; 2017.

20. Goicolea I, Hultstrand Ahlin C, Waenerlund A, Marchal B, Christianson M, Wiklund M, et al. Accessibility and factors associated with utilization of mental health services

For peer review only

For peer review only - http://bmjopen.bmj.com/site/about/guidelines.xhtml

in youth health centers. A qualitative comparative analysis in northern Sweden (Report). Int J Ment Health Syst. 2018;12(1):69.

21. Atun R, de Jongh T, Secci F, Ohiri K, Adeyi O. Integration of targeted health interventions into health systems: a conceptual framework for analysis. Health Policy Plan. 2009;25(2):104–11.

22. Ellis L, Churruca K, Braithwaite J. Mental health services conceptualised as complex adaptive systems: what can be learned? Int J Ment Health Syst 2017;11:43.

23. The health care act. Minstry of health. Hälso- och sjukvårdslagen. Svensk författningssamling 1982:76. Stockholm: Socialdepartementet.

24. National Board of Health and Welfare. Socialstyrelsen. (2009) Barn- och ungdomspsykiatri – vård på olika villkor. Rapport från en nationell tillsyn. Stockholm.

25. Pawson R. The science of evaluation: a realist manifesto. Thousand Oaks, CA: Sage; 2013.

26. Kane M, Trochim WM. Concept mapping for planning and evaluation. Thousand Oaks, CA: Sage; 2007.

27. Palinkas L, Aarons G, Horwitz S, Chamberlain P, Hurlburt M, Landsverk J. Mixed method designs in implementation research. Adm Policy Ment Health. 2011;38(1);44–53.

28. Marchal B, van Belle S, van Olmen J. Is realist evaluation keeping its promise? A review of published empirical studies in the field of health systems research. Evaluation. 2012;18(2):192–212.

29. Clarke V, Braun V. Thematic analysis. The journal of positive psychology. 2016;12(3):297–8.

For peer review only

For peer review only - http://bmjopen.bmj.com/site/about/guidelines.xhtml

30. Clark CD. The autodriven interview: a photographic viewfinder into children's experience. Vis Sociol. 1999;14(1):39–50.

31. Goicolea I, Hurtig A-K, San Sebastian M, Marchal B, Vives-Cases C. Using realist evaluation to assess primary healthcare teams' responses to intimate partner violence in Spain. Gaceta Sanitaria. 2015;29(6):431–6.

32. Trochim, W., & Kane, M. (2005). Concept mapping: an introduction to structured conceptualization in health care. International Journal for Quality in Health Care, 17(3), 187–191.

33. Brown, R., & Pehrson, S. (2019). Group Processes: Dynamics Within and Between Groups. John Wiley & Sons, Incorporated.

Figure 1 The Model for Mental Health Care Delivery as a Complex Adaptive System inspired by Ellis et al [22]

<Figure 2 Conceptual Framework to Analyze Integration of Mental Health Care for Youth within Swedish Youth Clinics>

For peer review only

For peer review only - http://bmjopen.bmj.com/site/about/guidelines.xhtml

Figure 1.

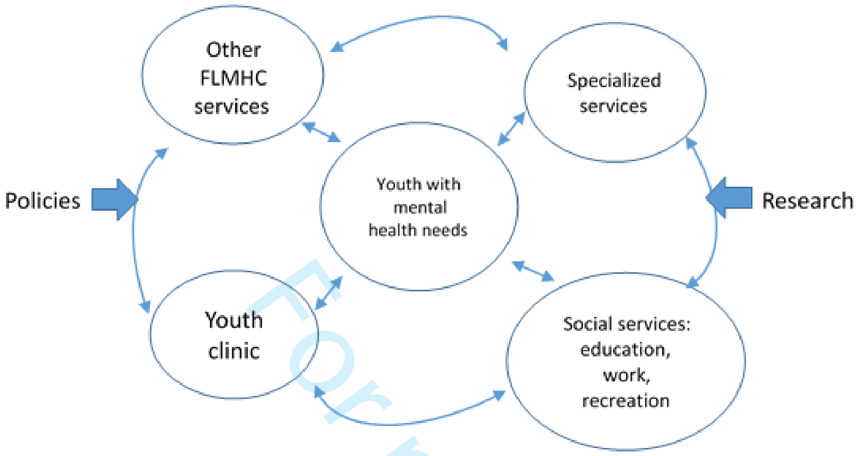

For peer review only

1 Figure 2

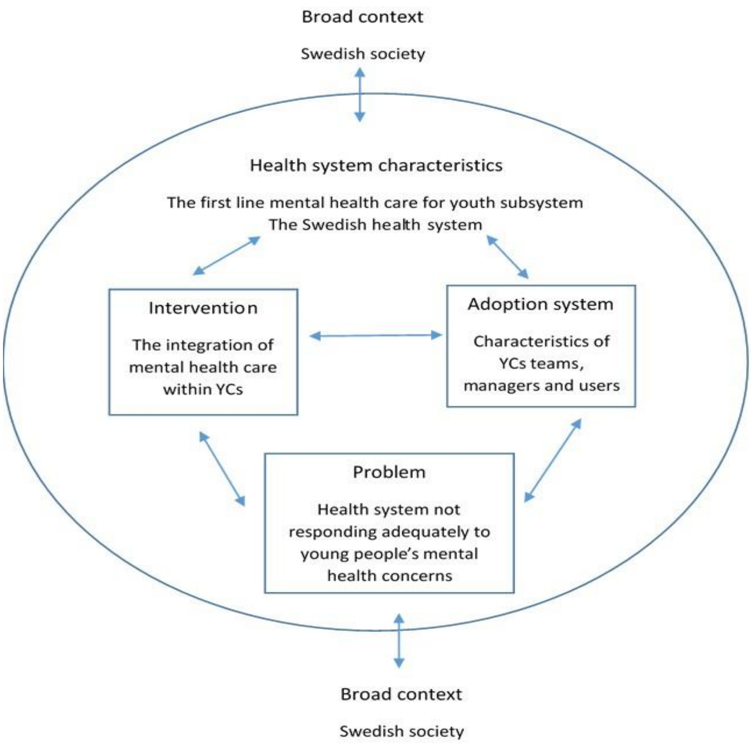

2

For peer review only

BMJ Open

How can we strengthen mental health services in Swedish youth clinics? A health policy and systems study protocol

Journal:	*BMJ Open*
Manuscript ID	bmjopen-2021-048922.R1
Article Type:	Protocol
Date Submitted by the Author:	22-Jun-2021
Complete List of Authors:	Richter Sundberg, Linda; Umea University, Department of Epidemiology and Global Health<br>Christiansson, Monica ; Umea University, Department of Nursing<br>Wiklund, Maria; Umea University, Department of Community Medicine and Rehabilitation<br>Hurtig, Anna-Karin ; Umea University, Department of Epidemiology and Global Health<br>Goicolea, I; Umea University, Department of Epidemiology and Global Health
**Primary Subject Heading**:	Mental health
Secondary Subject Heading:	Public health, Health policy, Health services research, Mental health, Research methods
Keywords:	MENTAL HEALTH, Organisation of health services < HEALTH SERVICES ADMINISTRATION & MANAGEMENT, Child & adolescent psychiatry < PSYCHIATRY, Health policy < HEALTH SERVICES ADMINISTRATION & MANAGEMENT

SCHOLARONE™
Manuscripts

I, the Submitting Author has the right to grant and does grant on behalf of all authors of the Work (as defined in the below author licence), an exclusive licence and/or a non-exclusive licence for contributions from authors who are: i) UK Crown employees; ii) where BMJ has agreed a CC-BY licence shall apply, and/or iii) in accordance with the terms applicable for US Federal Government officers or employees acting as part of their official duties; on a worldwide, perpetual, irrevocable, royalty-free basis to BMJ Publishing Group Ltd ("BMJ") its licensees and where the relevant Journal is co-owned by BMJ to the co-owners of the Journal, to publish the Work in this journal and any other BMJ products and to exploit all rights, as set out in our licence.

The Submitting Author accepts and understands that any supply made under these terms is made by BMJ to the Submitting Author unless you are acting as an employee on behalf of your employer or a postgraduate student of an affiliated institution which is paying any applicable article publishing charge ("APC") for Open Access articles. Where the Submitting Author wishes to make the Work available on an Open Access basis (and intends to pay the relevant APC), the terms of reuse of such Open Access shall be governed by a Creative Commons licence – details of these licences and which Creative Commons licence will apply to this Work are set out in our licence referred to above.

Other than as permitted in any relevant BMJ Author's Self Archiving Policies, I confirm this Work has not been accepted for publication elsewhere, is not being considered for publication elsewhere and does not duplicate material already published. I confirm all authors consent to publication of this Work and authorise the granting of this licence.

For peer review only

How can we strengthen mental health services in Swedish youth clinics? A health policy and systems study protocol

Linda Richter-Sundberg[1,*], Monica Christianson[2], Maria Wiklund[3], Anna-Karin Hurtig[1], Isabel Goicolea[1]

[1]Department of Epidemiology and Global Health Umeå University, Umeå, Sweden

[2]Department of Nursing, Umeå University, Umeå, Sweden

[3]Unit of Physiotherapy, Department of Community Medicine and Rehabilitation, Umeå University, Umeå, Sweden

*Correspondence: linda.sundberg@umu.se

Word count 5978

For peer review only

Abstract

Introduction

Strengthening first-line mental health care services for youth remains a priority for the Swedish government. The government is currently investigating how different sectors involved can be strengthened, but evidence is scarce. Youth clinics' play a key role in these discussions, being one of the most trusted services for youth. However, analysis of organizational functions and coordination with other services is important to strengthen youth-clinics role in first-line mental health care. This study investigates these challenges and aims to analyze the integration of mental health care within youth clinics to identify strategies to strengthen first-line mental health care for youth in Sweden.

Methods and analysis This study adopts a health policy and systems approach. In the first phase, a formative realist evaluation is conducted to ascertain what works in terms of integrating mental health care services within youth clinics, for what type of youth subpopulations, and under what circumstances. National-level stakeholders will be interviewed to elicit the program theory that explains how the intervention is supposed to work. The program theory will then be tested in 3-5 cases. The cases will be comprised by youth clinics and their stakeholders. Quantitative and qualitative information will be gathered, including via visual methodologies and questionnaires. The second phase includes a concept mapping study, engaging stakeholders and young people to build consensus on strategies to strengthen the integration of mental health care into youth clinics.

Ethics and dissemination The Swedish Ethical Review Authority has approved the study (2019-02910, 2020-04720). The results will be published in open-access peer-reviewed journals and presented at scientific conferences.

For peer review only - http://bmjopen.bmj.com/site/about/guidelines.xhtml

Keywords: Implementation, Mental health, Youth health systems, Primary health care, Integration, Youth clinic

Article Summary

Strengths and limitations

- The study addresses an urgent problem in health systems globally – the barriers to youth accessing high quality mental health services.
- The study will generate knowledge to strengthen first-line mental health care for youth in Sweden and in similar settings where differentiated services for youth that integrate mental health care are being developed.
- This study will add the team and organizational levels to the individual level, which might challenge the method of realist evaluation to some degree.
- A challenge of the study is to ensure meaningful participation of young people, especially in the concept mapping step of the study

Introduction

During the last decade, the increase of mental health problems among youth is evident in different parts of the world [1-2]. In parallel, there are signs of health systems struggling to adequately respond to youth mental health needs, in particular in implementing early interventions [3-4]. More than 75% of mental health problems have onset during childhood or adolescence, therefore this is a period of opportunity for health systems to promote youth mental health and respond to youth's mental health problems in coordination with other actors and sectors [5,6]. International research shows that access to mental health services for youths

For peer review only - http://bmjopen.bmj.com/site/about/guidelines.xhtml
For peer review only

is often hindered by factors such as long waiting times, youth concerns about confidentiality, and lack of trust in professionals [6, 7]. The transfer between youth mental health services (e.g., from child mental health services to adult mental health services) is experienced by young people as dramatic and with poor coordination [8, 9].

In Sweden, the prevalence of mental health problems among young people has increased dramatically with about 70% during the period 2006–2016, the highest increase among the Nordic countries. Depression, anxiety, and substance abuse have shown the largest increase [10, 11], and suicide rates have not decreased in younger age groups unlike in the overall population [12].

To enhance accessibility to mental health care services for youth and facilitate coordination, the Swedish health system since 2009 has been developing a strategy to integrate youth mental health services within primary care services. The integration is labeled First Line Mental Health Services (FLMHS) [13, 14]. This approach has been used in several other countries [15, 16]. Research has shown that the integration of mental health services within primary care improves the management of common mental health problems among adults [16, 17] and children [18]. Youth between 15 and 25 years of age have found it easier to access mental health services when delivered through less stigmatized, more visible and familiar primary care services [19]. Reforms of youth mental health services have been implemented in many countries, but surprisingly, there is still limited research analyzing integrated care models such as the FLMHS [19-21].

The FLMHS was launched as a way to address three main challenges: (1) inequities in the mental health and social services provided to children and youth, (2) unclear division of responsibilities within and between health and social services, and (3) a need to develop

For peer review only

For peer review only - http://bmjopen.bmj.com/site/about/guidelines.xhtml

versatile health system solutions reflecting the diverse conditions prevailing in the different groups of the population and parts of the country. The establishment of FLMHS was in addition expected to relieve the high (unfulfilled) demand for specialized child mental health services and strengthen the collaboration between diverse public services and sectors [22, 23]. Currently, the sectors that are most frequently involved in FLMHS are primary health care centers, school health services and youth clinics [24]. More than 250 youth clinics have been in operation in Sweden since the 1970-1980s, providing youth-centered health services for young people. This national network of YCs offers an important opportunity to integrate mental health services within services that report a high level of trust by young people [25]. Youth commonly approach YCs with needs and questions that falls within the area of SRH, e.g. requests for prescriptions for birth control pills, tests or treatment of sexually transmitted infections. The identity and role of YCs is well established among youth, YC health professionals and in other parts of the health system. However, ever since their establishment in the 1970s, YCs are also responding to youth needs in regards to mental health and mental health problems. YC has come to play a key role in the FLMHS where youth mental health services are integrated in primary care level. YCs operate together with other stakeholders within the FLMHS and interacts with stakeholders from the larger youth mental health system, e.g. authorities, specialized health services and schools (fig. 1).

<Figure 1 Stakeholders in and surrounding the FLMHS for youth >

In this paper, we present the protocol for a health policy and system study with an aim to analyze Swedish policy efforts to strengthen FLMHS for youth. We focus on YCs and adopt Atun's et al conceptual framework for investigating the integration of health interventions

For peer review only - http://bmjopen.bmj.com/site/about/guidelines.xhtml

into health systems [26]. The research questions we aim to answer with this study are as follows: (1) How and under what circumstances are YCs part of the FLMHS for youth in the Swedish health system? (2) How can the integration of mental health services into YCs in the Swedish health system be strengthened?

Conceptual framework

The approach to mental health systems used in this study is inspired by complexity theory [27], with a specific focus on the integration of targeted health interventions within health systems [26]. Instead of portraying the different hierarchical levels and actors in the mental health system [22], complexity theory portrays them as interconnected and centered on the service user (fig. 2). In complex adaptive systems, individuals, services, and organizations are active agents that interact with each other, establishing dynamic interconnections [27].

<Figure 2 The Model for Mental Health Care Delivery as a Complex Adaptive System inspired by Ellis et al [27].>

This study will explore the intentions and strategies among national stakeholders and policy makers (e.g., authorities, politicians) in terms of integrating YCs in FLMHS and if and how YCs coordinate with other health actors in the FLMHS for youth, with specialized mental health services and with social services. The organization of this system is influenced by, and influences, both policies and research (Fig. 2).

Inspired by Atun et al. [26], we conceive integration as the process, pattern, and extent of adoption and eventual assimilation of new strategies into the health system (in this case, mental health services into YCs) that are embedded within FLMHS for youth.

For peer review only - http://bmjopen.bmj.com/site/about/guidelines.xhtml

In Figure 3, we have adapted Atun et al.'s framework to the specific intervention being analyzed in this study. Atun et al.'s conceptual framework provides a systematic approach to researching integration by focusing on 5 domains that influence the adoption and diffusion of health interventions.

<Figure 3 Conceptual Framework to Analyze Integration of Mental Health Care for Youth within Swedish Youth Clinics>

The first domain relates to the nature of the problem being addressed, i.e., the understanding regarding the urgency and severity of the problem influences the speed of adoption [26]. In this protocol, we conceptualize the problem as the weaknesses of the health system to adequately respond to the increasing mental health needs of young people.

The second domain refers to the characteristics of the intervention. Here, Atun et al. point out that interventions that for example are perceived as having a relative advantage, are less complex and risky and are more easily integrated. In this protocol, the intervention to address this problem is the integration of mental health services within the existing network of Swedish YCs.

The third domain refers to the adoption system, namely the key actors and institutions involved in the integration [26]. Each stakeholder has differing perceptions of the benefits, risks, and legitimacy of the intervention, and the different interests at stake have to be explored. The adoption system in this study focuses on the YC care providers, the managers, and the young service users.

The fourth domain refers to the characteristics of the health system [26]. With regard to the characteristics of the health system, we are interested in the larger mental health system (i.e.,

For peer review only

national governance, primary and specialized mental health and social services), but with special focus on the sub-system of FLMHS for youth. The focus is on analyzing the collaboration between the services responsible for FLMHS for youth and how the different ways of organizing them and their approaches toward young people influence both the integration of mental health services within YCs and the collaborative dynamics with the other services.

The fifth domain refers to the broader socio-cultural context, where critical events, prevailing political economy, and socio-cultural norms also affect integration [26]. The broader context in the study protocol refers to how the current political economic situation and the socio-cultural characteristics of Swedish society, influence the integration of mental health services for youth within YCs.

Material and methods

Study design

To address our two main research questions, two methodological approaches will be combined in a sequential way: (1) a realist evaluation [28] and (2) concept mapping [29]. The study design will follow an iterative analytical process in which the conduct of the concept mapping will draw on the findings from the realist evaluation. Qualitative and quantitative data collection and analysis will be combined to best capture the complexity of the phenomenon under investigation. The integration of qualitative and quantitative approaches enables researchers to explore phenomena from different perspectives and provides an enriched understanding [30]. The data collection started in November 2019 and will be finalized in November 2022. The main research questions and the methodologies applied are summarized in Table 1.

For peer review only - http://bmjopen.bmj.com/site/about/guidelines.xhtml

Table 1: The research process

Research question in focus	Methodological approach	Steps	Methods for data collection	Methods for data analysis
How and under what circumstances are YCs part of the FLMHS for youth in the Swedish health care system?	Realist evaluation	Eliciting the program theory	Literature review	
			Documentary review	Thematic analysis
			Interviews with stakeholders	
		Testing the program theory in selected cases	Analytical multiple case study	
			Questionnaires for mental health care consultations	Descriptive statistical analysis
			Interviews with professionals from YCs	Thematic analysis
		Refining the program theory	Interviews with professionals from other services involved in FLMHS for youth	
			Interviews with professionals at specialized services	Thematic analysis
			Photo-elicitation with young people	
How can the integration of mental health care services into YCs in the Swedish health system be strengthened?	Concept mapping	Presenting the findings and engaging professionals and young people	Workshop and photo exhibition with (1) professionals working in YCs, (2) professionals in other FLMHS services for youth and in managerial positions, and (3) young people and youth organizations	
		Developing the concept maps	Brainstorming, pile sorting and rating, and map development using Concept System-CS Global Max	Concept mapping techniques (mixed methodology combining multivariate statistical methods, and collective qualitative interpretation)
		Bringing everything together and proposing actions	Final workshop	

For peer review only

Patient and public involvement

The research questions in this study have been informed by the experiences and preferences of youth, through our previous studies exploring youth views on YCs. Furthermore, the study adopts a participatory approach which has implications for the design, conduct of the study and also the choice of outcome measures.

For peer review only - http://bmjopen.bmj.com/site/about/guidelines.xhtml

All phases of the study will be presented and discussed with stakeholders at youth clinics (both managers and professionals) and other arenas (i.e. youth organizations) before and during data collection. Dissemination is a core component of the second phase. Stakeholders (including young people) will be invited to workshops to discuss preliminary results and engage in a concept mapping to collectively develop a set of strategies to improve the integration of mental health services within YCs. One of the main outcomes in this study is to reach consensus on the strategies to strengthen the integration of youth clinics in the FLMHS.

Study setting

The responsibility for providing health services in the Swedish health care system is shared between the national government, the regions, and the municipalities. The Swedish Health Care Act gives a commitment to provide "good health and care on equal terms for the entire population" [31]. However, evaluations have shown that evidence-based health services are inaccessible to children and youth with mental health problems to an extent that diverges from the Health Care Act [32].

Data collection and analysis phase I - Formative realist evaluation

To answer the first research question, a formative realist evaluation of the integration of mental health services within YCs will be conducted. Realist evaluation is well suited to analysis of complex interventions aiming to ascertain whether the intervention works but also why, for whom, and under what circumstances [28]. In realist evaluation, theories are at the middle-range level, connecting the intervention with contextual factors, with the mechanisms that the intervention triggers within the target populations, and with the outcomes achieved. The aim is to identify patterns of intervention-context-mechanism-outcomes that can explain how, why, and under what circumstances a specific intervention works.

For peer review only - http://bmjopen.bmj.com/site/about/guidelines.xhtml
For peer review only

Pawson [28] describes cycles of realist evaluation, starting from eliciting the program theory behind the implementation of a particular intervention, followed by putting this theory under test in specific cases and coming up with a refined program theory. The entire process should be conducted in close dialogue with stakeholders involved in the development and implementation of the intervention, because the aim is to come up with a revised and improved intervention [28].

Realist evaluation can be applied in a summative way (once an intervention has ended or come to a conclusion) or in a formative way (while an intervention is being implemented). Integrated care programs for young people's mental health need to adapt and learn continuously, therefore we view our realist evaluation through the lens of formative evaluations, aiming for continuous quality improvement. The realist evaluation will cover the following steps: (1) developing the program theory, (2) testing the program theory in selected cases, (3) refining the program theory.

Developing the program theory

The realist evaluation will start developing the program theory through a review of the literature and policy documents. The scientific literature, policies, and other relevant documents related to the intervention will be reviewed, looking for key concepts, ideas, and theories that can help to explain how the integration of mental health services for youth within the FLMHS is supposed to work. The scientific literature and documents will be analyzed using thematic analysis following Clarke & Braun [33].

In addition, we will interview national- and regional-level stakeholders involved in developing the intervention (i.e., the Swedish Association of Local Authorities and Regions, The Swedish Association of Youth Clinics). The interviews will aim to make the program theory behind the intervention explicit, namely the integration of mental health services within YCs, and to analyze how the increasing problems of youth mental health and the

For peer review only - http://bmjopen.bmj.com/site/about/guidelines.xhtml

planned solutions/responses are understood. The five domains of Atun et al.'s framework [26] in relation to the three key concepts of realist evaluation (context, mechanisms, outcomes) will be the main topics explored. The interviews will be audio-recoded, transcribed, and analyzed using abductive thematic analysis [33], moving back and forward between our conceptual framework and the data. The program theory that develops will explain how the intervention is supposed to work. Following the ideas of FLMHS [22], the outcomes will mainly focus on whether the YCs (1) are accessible, (2) allow early detection, (3) have broad expertise of good quality, (4) have good reporting and monitoring systems, and (5) have good collaboration with other services [22].

Testing the program theory in selected cases

In the next phase, the program theory will be put to the test in 3–5 specific cases: YCs and the FLMHS in which they are embedded, including the surrounding referral services. To capture diverse contextual circumstances, we will choose clinics in different municipalities, located in different regions, and embedded in different models of FLMHS for youth (as defined by the Swedish Association of Local Authorities and Regions [22]).

From each case, quantitative information will be gathered on the extent and characteristics of mental health consultations. For this study, we will distribute the questionnaire to all the professionals working at the YC in the 3–5 cases, including midwives, psychologists, social workers, medical doctors, and other staff who are frequently approached by young people to discuss mental health issues. The questionnaire will be applied over 6 months in each of the clinics participating as cases. Analysis of the quantitative data will offer answers whether the services (1) are accessible, (2) allow early detection, and (3) offer good quality services [23].

Qualitative information from each case will include interviews (~5–10 per case) with (1) professionals working in the YC, (2) professionals working in other FLMHS for youth (e.g., student health, health centers, social services), and (3) professionals working in specialized

For peer review only - http://bmjopen.bmj.com/site/about/guidelines.xhtml

mental health services. Because the way different municipalities are implementing FLMHS for youth and the services with responsibility for this sub-system vary, the institutions represented might vary from case to case. Professionals working in the YCs will include the head of the clinic and a variety of professionals, including professions more focused on mental health (e.g., counselor, psychologist) and others (e.g., midwives). Interviews at this stage will focus on health professionals' experiences of mental health consultations and if and how mental health services have been integrated and possibly sustained within the clinic's practice, coordination within the clinic and with other health care resources (both first-line and specialized) and social resources. Professionals working in FLMHS for youth will include those working in, for example, primary health care centers and school health services. Specialist mental health professionals will include those working in child mental health services and adult mental health services. Interviews with staff in specialized mental health services will focus on how they perceive the role of YCs in FLMHS for youth, and their collaboration with YCs and with other services. Data from the interviews will be analyzed using thematic analysis [33].

To capture the views and experiences of young people (aged 16–29 years), we will engage young people in an auto-driven photo-elicitation interview to gather their perspectives [34]. Photo-elicitation is a research method that incorporates images in the interview process to elicit participants' subjective explanations. Photo-elicitation has gained recognition as a way to better capture young people's attention, ease rapport, and balance power differentials.

From each case, 6–8 youths aged 16–29 years will be recruited. Participants will include youth with experience using YC mental health services and those who do not have that experience. For the latter, we will recruit young people from high schools and universities. First, each participant will be approached and receive information about the study, offered a digital camera for 2 months and asked to take pictures to depict young people experiences

For peer review only - http://bmjopen.bmj.com/site/about/guidelines.xhtml
For peer review only

and/or expectations of mental health and/or mental health services in the area where they live. At a second meeting, the participant will select 3 of the pictures taken as the basis for an interview, where topics around youth mental health, access to services, and experiences of them (with special focus on YCs) will be discussed.

All the interviews will be audio-recorded and transcribed. Detailed descriptions of the cases will be developed and further analyzed using an abductive thematic analysis approach, guided by the framework presented above (fig. 2). This analysis will inform the intervention implementation, contextual factors, potential mechanisms, and outcomes.

Refining the program theory

The initial program theory will be refined based on the findings in the theory testing to specify a middle-range theory that describes what works in terms of integrating mental health services for young people within YCs, for what type of youth subpopulations, and under what contextual circumstances [28, 35].

Data collection and analysis phase II – A concept mapping study

Building upon the knowledge gained during the previous phase, we will engage in a participatory process in which professionals and young people will be invited to take part in workshops followed by a concept mapping study [29]. The aim of this study is to develop strategies to strengthen the integration of mental health services within YCs. The concept mapping entails three steps: presenting findings and engaging professionals and young people in participatory workshops, developing the concept maps, and bringing everything together and proposing actions.

For peer review only - http://bmjopen.bmj.com/site/about/guidelines.xhtml

Presenting findings and engaging professionals and young people in participatory workshops

To present the preliminary results of the study, workshops will be arranged with 3 different types of stakeholders: (1) professionals working in YCs, (2) professionals in other FLMHS for youth and in managerial positions, and (3) young people and youth organizations. Youth will be recruited to the workshop using three strategies: first, the youth who were involved in the previous interview study in the project will be asked to participate in the workshop; second, YCs involved in the case studies will be asked to invite youth through advertising in waiting rooms/reception areas; and third, youth delegations will be asked to nominate youth to participate in the workshop.

Developing the concept maps

Using the findings from the participatory workshops as a basis for reflection, participants will be asked to participate in a concept mapping study to collectively develop a set of strategies to improve the integration of mental health services within YCs.

Concept mapping enables groups of actors to visualize their ideas about an issue of mutual interest and to develop common frameworks through a structured, participatory process. Qualitative and quantitative data are generated and integrated by participants through sequential steps, beginning with the generation of ideas (brainstorming), structuring of ideas through sorting and rating, development of conceptual maps based on multivariate statistical methods, and collective interpretation of the maps [29]. Data analysis will adopt concept mapping techniques with a mixed methodology combining multivariate statistical methods, and collective qualitative interpretation.

The research team will develop a focus question to guide the brainstorming activity and with clear instructions to all workshop participants and other relevant stakeholders and young

For peer review only - http://bmjopen.bmj.com/site/about/guidelines.xhtml

For peer review only

people. Once the refined list of actions is developed, it will be sent again to the participants for sorting and rating. Three different sets of maps (one for each group) will be developed. The data will be analyzed using concept mapping techniques, which facilitate visualization of thematic clusters and identification of areas of consensus for action. The Concept System-CS Global Max software will be used throughout the process.

Bringing everything together and proposing actions

Finally, the concept maps and all the qualitative and quantitative results will be synthesized to answer our main research question: "How can the integration of mental health services into YCs in the Swedish health system be strengthened?" Building on the concept maps and synthesized results, a final workshop will be held with young people and relevant stakeholders to discuss the maps and come up with a set of elaborated strategies.

Ethics and dissemination

This study has received approval from the Swedish Ethical Review Agency (Dnr 2019-2910, 2020-04720) and complies with the Helsinki Declaration. All participants will be informed that participation in the study is voluntary. Individuals will be provided with information in verbal and written format, and offered the opportunity to ask any questions. Written informed consent will be obtained from all participants.

Results will be published in open-access peer-reviewed journals and presented at scientific conferences, and as described before, preliminary findings will be discussed with stakeholders during the concept mapping.

For peer review

Discussion

This paper presents a study protocol using a multiple case-study design to understand the integration of mental health services within YCs with a view to strengthening FLMHS for youth in Sweden and to develop strategies for strengthening this process.

This study is timely as it goes hand in hand in informing policy efforts to integrate mental health services within YCs and YCs within the FLMHS for youth in Sweden. Atun et al.'s [26] framework has been used to analyze the integration of programs on communicable diseases, cardiovascular disease, and sexual and reproductive health interventions, but, to our knowledge, it has never been used to analyze the integration of mental health services in primary care. The methodological approach, involving realist evaluation and concept mapping, combines quantitative and qualitative methods sequentially in a way that fits the research questions and the health systems approach to complexity, and is innovative in the field of public health.

The study combines two methodological approaches: realist evaluation [28] and concept mapping [29]. Unlike randomized controlled trials, realist evaluation does not aim to control contextual factors but rather to explore how outcomes of an intervention interact with the context in which it is implemented. Realist evaluation is a methodology that rests on critical realism, and it mainly focuses on individual mechanisms in understanding an intervention. In this study, we do not focus only at the individual level but also the team and organizational levels. This challenges the method to some degree, but we have good experiences of using the method in this broader sense, for example, in the context of primary health care services [36].

Concept mapping is a mixed participatory method for involving stakeholders and youth in a structured conceptualization process. It has been developed to collect and integrate input from multiple sources and stakeholders with various demand, expertise or interest [37]. One

For peer review only - http://bmjopen.bmj.com/site/about/guidelines.xhtml

challenge in using concept mapping is that the methodology utilizes group processes, for example, in brainstorming activities. Interaction within groups and the group processes are affected by entities such as power and status [38]. As participants are diverse in terms of age, education, and gender, we see a potential risk that participants not will have equal possibilities to contribute in the concept mapping. To balance participant contributions in group-oriented tasks, we will have this challenge in mind as we put the groups together. The moderator of the session will also be instructed to facilitate the participation of all group members. By taking these measures, we hope to balance the potential negative impact of the group processes to some degree.

The results of the study are expected to contribute new knowledge concerning the process of integration of YC in the FLMHS for youth but also to lead to recommendations for policy makers and decision makers within the field of youth mental health services.

Covid-19 pandemic: adjustments

Due to the Covid-19 pandemic some adjustments to the planned methodology has been, and will be necessary. During year 2020 and 2021 meetings and travels has been restricted in Sweden, with the purpose of minimizing spread of the Covid-19 infection. The restrictions has implications for data collection, all interviews during this time were and will be performed digitally. The pandemic also brought an increased strain on the health system in all levels, affecting the possibility to recruit participants operating within the health system, for example in government authorities or in youth clinics. This has and will be handled by adjusting the timeline forward and being flexible in terms of how interviews and workshops will be carried out (i.e. digital or face-to face).

For peer review only - http://bmjopen.bmj.com/site/about/guidelines.xhtml

Authors' contributions

IG designed the research proposal, drafted the application, and led the discussion in the group as principal investigator of the research proposal. LRS led the development of the first draft of the manuscript based on the research proposal and gathered comments from all team members to revise successive drafts and develop and submit the final draft of the manuscript to the journal. IG, MC, MW, and AKH are part of the research team that supported and developed the protocol. They contributed with ideas and discussed the protocol that was submitted and granted funding from FORTE. They read and critically commented on and revised successive drafts of the manuscript and approved the final version before submission to the journal.

Funding statement

This work was supported by the Swedish Research Council for Working Life and Living Conditions (FORTE) grant number 2018-00364.

Competing interests

The authors declare that they have no competing interests.

For peer review only - http://bmjopen.bmj.com/site/about/guidelines.xhtml

References

1. Bitsko RH, Holbrook JR, Ghandour RM et al (2018) Epidemiology and impact of health care provider-diagnosed anxiety and depression among US children. J Dev Behav Pediatr 39(5):395–403.

2. Pitchforth J, Fahy K, Ford T, Wolpert M, Viner RM, Hargreaves DS. Mental health and well-being trends among children and young people in the UK, 1995–2014: analysis of repeated cross-sectional national health surveys. Psychological Medicine. Cambridge University Press; 2019;49(8):1275–85.

3. Rickwood DJ, Deane FP, Wilson CJ. When and how do young people seek professional help for mental health problems? Med J Aust. 2007 Oct 1;187(S7):S35-9.

4. Mcgorry, PD; Mei, Cristina. Early intervention in youth mental health: progress and future directions. Evidence-based mental health, 2018, 21.4: 182-184.

5.Weinberger A, Gbedemah M, Martinez A, Nash D, Galea, S, Goodwin R. Trends in depression prevalence in the USA from 2005 to 2015: widening disparities in vulnerable groups. Psychol Med. 2018;48(8):1308–15.

6. Patel V, Flisher AJ, Hetrick S, McGorry P. Mental health of young people: a global public-health challenge. Lancet. 2007;369(9569):1302–13.

7. Malla A, Iyer S, McGorry P, Cannon M, Coughlan H, Singh S, et al. From early intervention in psychosis to youth mental health reform: a review of the evolution and transformation of mental health services for young people. Soc Psychiatry Psychiatr Epidemiol. 2016;51(3):319–26.

For peer review only

For peer review only - http://bmjopen.bmj.com/site/about/guidelines.xhtml

8. Gulliver A, Griffiths KM, Christensen H. Perceived barriers and facilitators to mental health help-seeking in young people: a systematic review. BMC Psychiatry 2010;10(1):113.

9. Broad KL, Sandhu VK, Sunderji N, Charach A. Youth experiences of transition from child mental health services to adult mental health services: a qualitative thematic synthesis. BMC Psychiatry. 2017;17(1):380–11.

10. Bremberg S. Mental health problems are rising more in Swedish adolescents than in other Nordic countries and the Netherlands. Acta Paediatr. 2015;104(10):997–1004.

11. Calling, S., Midlöv, P., Johansson, SE. et al. Longitudinal trends in self-reported anxiety. Effects of age and birth cohort during 25 years. BMC Psychiatry 2017;17, 119

12. National Board of Health and Welfare (Socialstyrelsen). Utvecklingen av psykisk ohälsa bland barn och unga vuxna till och med 2016. Stockholm: Socialstyrelsen; 2017.

13. Swedish Association of Local Authorities and Regions (Sveriges Kommuner och Regioner). Uppdrag Psykisk Hälsa. Första linjen för barn och ungas psykiska hälsa. Stockholm: SALAR; 2016.

14. Swedish Association of Local Authorities and Regions (Sveriges Kommuner och Regioner). Uppdrag Psykisk Hälsa. Första linje boken. Stockholm: SALAR; 2017.

15. World Health Organization. Integrating mental health into primary care: a global perspective. Geneva: World Health Organization; 2008.

16. Druss BG, Goldman HH. Integrating Health and Mental Health Services: A Past and Future History. American Journal of Psychiatry. 2018 Dec 1;175(12):1199–204.

For peer review only - http://bmjopen.bmj.com/site/about/guidelines.xhtml

17. Kates N, Arroll B, Currie E, Hanlon C, Gask L, Klasen H, et al. Improving collaboration between primary care and mental health services. The World Journal of Biological Psychiatry. 2019 Nov 26;20(10):748–65.

18. Campo JV, Geist R, Kolko DJ. Integration of pediatric behavioral health services in primary care: improving access and outcomes with collaborative care. Can J Psychiatry. 2018;63(7):432–38.

19. Hetrick SE, Bailey AP, Smith KE, Malla A, Mathias S, Singh SP, et al. Integrated (one-stop shop) youth health care: best available evidence and future directions. Med J Aust 2017;207(S10):S5–18.

20. Kinchin I, Tsey K, Heyeres M, et al. Systematic review of youth mental health service integration research. Aust J Prim Health. 2016;22(4):304–15.

21. Richardson LP, McCarty CA, Radovic A, Suleiman AB. Research in the integration of behavioral health for adolescents and young adults in primary care settings: a systematic review. J Adolescent Health. 2016;60(3):261–9.

22. Swedish Association of Local Authorities and Regions (Sveriges Kommuner och Regioner). Psykisk hälsa på ungdomsmottagningen – en kartläggning av Sveriges ungdomsmottagningar. Stockholm: SALAR; 2017.

23. National Board of Health and Welfare (Socialstyrelsen). Kartläggning av första linjens verksamhet för barn och unga med psykisk ohälsa. Stockholm: Socialstyrelsen; 2021.

24. Swedish Association of Local Authorities and Regions (Sveriges Kommuner och Regioner). Första linjen 2020 Nulägesbeskrivning av hälso- och sjukvårdens första linje för barn och ungas psykiska hälsa. Stockholm: SALAR; 2020

For peer review only

25. Goicolea I, Hultstrand Ahlin C, Waenerlund A, Marchal B, Christianson M, Wiklund M, et al. Accessibility and factors associated with utilization of mental health services in youth health centers. A qualitative comparative analysis in northern Sweden (Report). Int J Ment Health Syst. 2018;12(1):69.

26. Atun R, de Jongh T, Secci F, Ohiri K, Adeyi O. Integration of targeted health interventions into health systems: a conceptual framework for analysis. Health Policy Plan. 2009;25(2):104–11.

27. Ellis L, Churruca K, Braithwaite J. Mental health services conceptualised as complex adaptive systems: what can be learned? Int J Ment Health Syst 2017;11:43.

28. Pawson R. The science of evaluation: a realist manifesto. Thousand Oaks, CA: Sage; 2013.

29. Kane M, Trochim WM. Concept mapping for planning and evaluation. Thousand Oaks, CA: Sage; 2007

30. Palinkas L, Aarons G, Horwitz S, Chamberlain P, Hurlburt M, Landsverk J. Mixed method designs in implementation research. Adm Policy Ment Health. 2011;38(1);44–53.

31. The health care act. Minstry of health. Hälso- och sjukvårdslagen. Svensk författningssamling 1982:76. Stockholm: Socialdepartementet.

32. National Board of Health and Welfare. Socialstyrelsen. (2009) Barn- och ungdomspsykiatri – vård på olika villkor. Rapport från en nationell tillsyn. Stockholm.

33. Clarke V, Braun V. Thematic analysis. The journal of positive psychology. 2016;12(3):297–8.

For peer review only

For peer review only - http://bmjopen.bmj.com/site/about/guidelines.xhtml

34. Clark CD. The autodriven interview: a photographic viewfinder into children's experience. Vis Sociol. 1999;14(1):39–50.

35. Marchal B, van Belle S, van Olmen J. Is realist evaluation keeping its promise? A review of published empirical studies in the field of health systems research. Evaluation. 2012;18(2):192–212.

36. Goicolea I, Hurtig A-K, San Sebastian M, Marchal B, Vives-Cases C. Using realist evaluation to assess primary healthcare teams' responses to intimate partner violence in Spain. Gaceta Sanitaria. 2015;29(6):431–6.

37. Trochim, W., & Kane, M. (2005). Concept mapping: an introduction to structured conceptualization in health care. International Journal for Quality in Health Care, 17(3), 187–191.

38. Brown, R., & Pehrson, S. (2019). Group Processes: Dynamics Within and Between Groups. John Wiley & Sons, Incorporated.

For peer review only

For peer review only - http://bmjopen.bmj.com/site/about/guidelines.xhtml

Figure 1 Stakeholders in and surrounding the FLMHS for youth

Figure 2 The Model for Mental Health Care Delivery as a Complex Adaptive System inspired by Ellis et al [22]

Figure 3 Conceptual Framework to Analyze Integration of Mental Health Care for Youth within Swedish Youth Clinics

For peer review only

For peer review only - http://bmjopen.bmj.com/site/about/guidelines.xhtml

Figure 1.

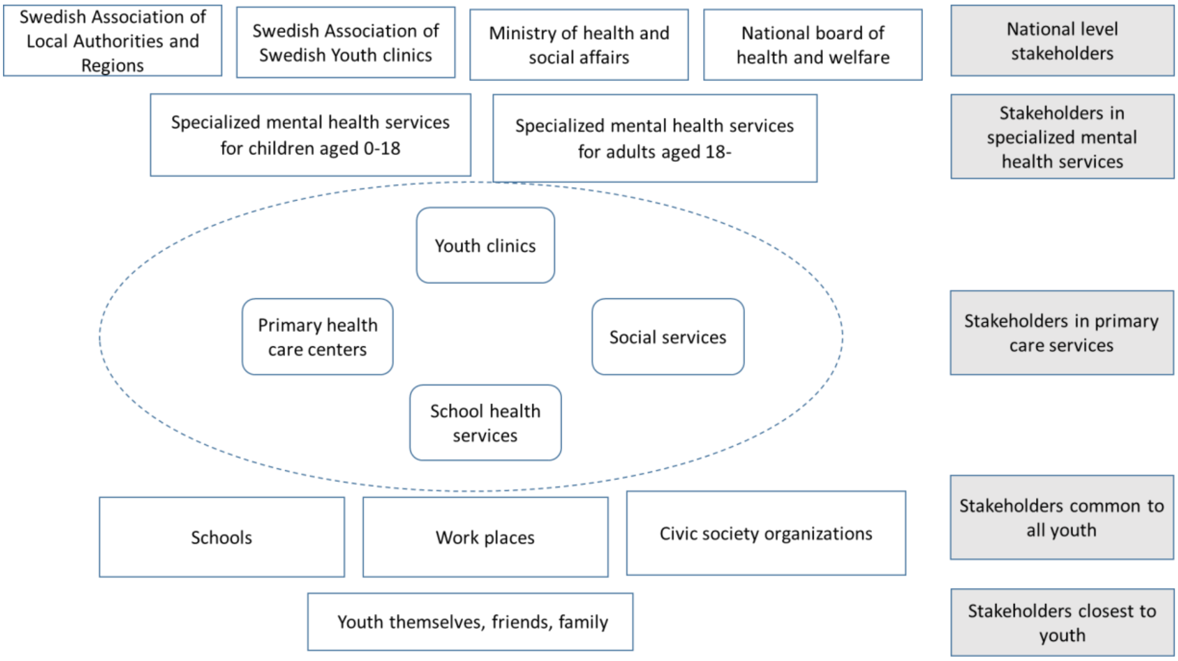

For peer review only - http://bmjopen.bmj.com/site/about/guidelines.xhtml

Figure 2.

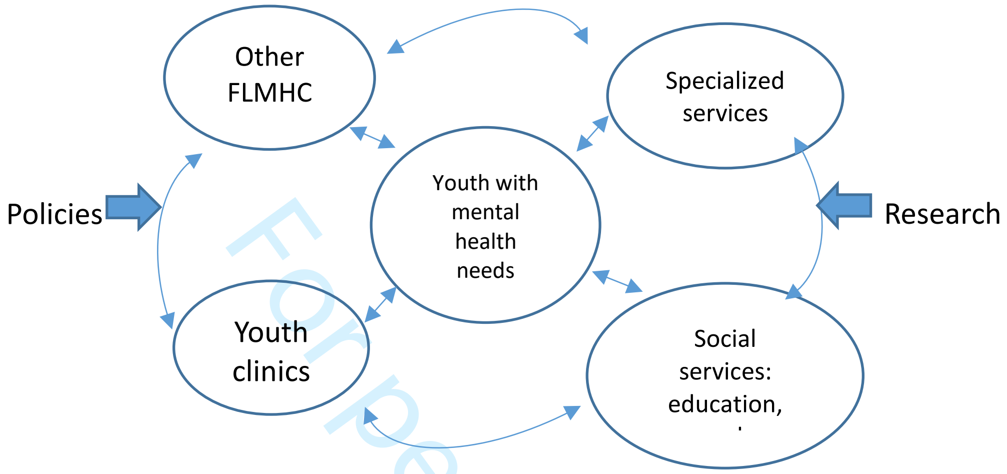

For peer review only

Figure 3

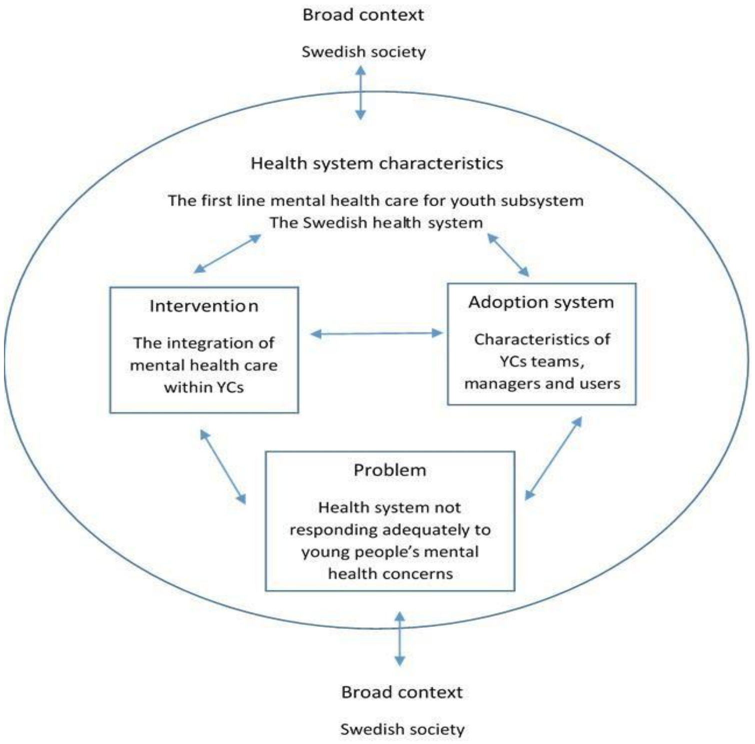

review only

For peer review only - http://bmjopen.bmj.com/site/about/guidelines.xhtml

BMJ Open

How can we strengthen mental health services in Swedish youth clinics? A health policy and systems study protocol

Journal:	*BMJ Open*
Manuscript ID	bmjopen-2021-048922.R2
Article Type:	Protocol
Date Submitted by the Author:	05-Oct-2021
Complete List of Authors:	Richter Sundberg, Linda; Umea University, Department of Epidemiology and Global Health<br>Christiansson, Monica ; Umea University, Department of Nursing<br>Wiklund, Maria; Umea University, Department of Community Medicine and Rehabilitation<br>Hurtig, Anna-Karin ; Umea University, Department of Epidemiology and Global Health<br>Goicolea, I; Umea University, Department of Epidemiology and Global Health
**Primary Subject Heading**:	Mental health
Secondary Subject Heading:	Public health, Health policy, Health services research, Research methods
Keywords:	MENTAL HEALTH, Organisation of health services < HEALTH SERVICES ADMINISTRATION & MANAGEMENT, Child & adolescent psychiatry < PSYCHIATRY, Health policy < HEALTH SERVICES ADMINISTRATION & MANAGEMENT

SCHOLARONE™
Manuscripts

I, the Submitting Author has the right to grant and does grant on behalf of all authors of the Work (as defined in the below author licence), an exclusive licence and/or a non-exclusive licence for contributions from authors who are: i) UK Crown employees; ii) where BMJ has agreed a CC-BY licence shall apply, and/or iii) in accordance with the terms applicable for US Federal Government officers or employees acting as part of their official duties; on a worldwide, perpetual, irrevocable, royalty-free basis to BMJ Publishing Group Ltd ("BMJ") its licensees and where the relevant Journal is co-owned by BMJ to the co-owners of the Journal, to publish the Work in this journal and any other BMJ products and to exploit all rights, as set out in our licence.

The Submitting Author accepts and understands that any supply made under these terms is made by BMJ to the Submitting Author unless you are acting as an employee on behalf of your employer or a postgraduate student of an affiliated institution which is paying any applicable article publishing charge ("APC") for Open Access articles. Where the Submitting Author wishes to make the Work available on an Open Access basis (and intends to pay the relevant APC), the terms of reuse of such Open Access shall be governed by a Creative Commons licence – details of these licences and which Creative Commons licence will apply to this Work are set out in our licence referred to above.

Other than as permitted in any relevant BMJ Author's Self Archiving Policies, I confirm this Work has not been accepted for publication elsewhere, is not being considered for publication elsewhere and does not duplicate material already published. I confirm all authors consent to publication of this Work and authorise the granting of this licence.

For peer review only

How can we strengthen mental health services in Swedish youth clinics? A health policy and systems study protocol

Linda Richter-Sundberg[1,*], Monica Christianson[2], Maria Wiklund[3], Anna-Karin Hurtig[1], Isabel Goicolea[1]

[1]Department of Epidemiology and Global Health Umeå University, Umeå, Sweden

[2]Department of Nursing, Umeå University, Umeå, Sweden

[3]Unit of Physiotherapy, Department of Community Medicine and Rehabilitation, Umeå University, Umeå, Sweden

*Correspondence: linda.sundberg@umu.se

Word count 5978

For peer review only

Abstract

Introduction

Strengthening first-line mental health care services for youth remains a priority for the Swedish government. The government is currently investigating how different sectors involved can be strengthened, but evidence is scarce. Youth clinics' play a key role in these discussions, being one of the most trusted services for youth. However, analysis of organizational functions and coordination with other services is important to strengthen youth-clinics role in first-line mental health care. This study investigates these challenges and aims to analyze the integration of mental health care within youth clinics to identify strategies to strengthen first-line mental health care for youth in Sweden.

Methods and analysis This study adopts a health policy and systems approach. In the first phase, a formative realist evaluation is conducted to ascertain what works in terms of integrating mental health care services within youth clinics, for what type of youth subpopulations, and under what circumstances. National-level stakeholders will be interviewed to elicit the program theory that explains how the intervention is supposed to work. The program theory will then be tested in 3-5 cases. The cases will be comprised of youth clinics and their stakeholders. Quantitative and qualitative information will be gathered, including via visual methodologies and questionnaires. The second phase includes a concept mapping study, engaging stakeholders and young people to build consensus on strategies to strengthen the integration of mental health care into youth clinics.

Ethics and dissemination The Swedish Ethical Review Authority has approved the study (2019-02910, 2020-04720). The results will be published in open-access peer-reviewed journals and presented at scientific conferences.

For peer review only

For peer review only - http://bmjopen.bmj.com/site/about/guidelines.xhtml

Keywords: Implementation, Mental health, Youth health systems, Primary health care, Integration, Youth clinic

Article Summary

Strengths and limitations

- The study addresses an urgent problem in health systems globally – the barriers to youth accessing high quality mental health services.
- The study will generate knowledge to strengthen first-line mental health care for youth in Sweden and in similar settings where differentiated services for youth that integrate mental health care are being developed.
- This study will add the team and organizational levels to the individual level, which might challenge the method of realist evaluation to some degree.
- A challenge of the study is to ensure meaningful participation of young people, especially in the concept mapping step of the study

Introduction

During the last decade, the increase of mental health problems among youth is evident in different parts of the world [1-2]. In parallel, there are signs of health systems struggling to adequately respond to youth mental health needs, in particular when implementing early interventions [3-4]. More than 75% of mental health problems have onset during childhood or adolescence, therefore this is a period of opportunity for health systems to promote youth mental health and respond to youth's mental health problems in coordination with other actors and sectors [5,6]. International research shows that access to mental health services for youths

For peer review only - http://bmjopen.bmj.com/site/about/guidelines.xhtml

is often hindered by factors such as long waiting times, youth concerns about confidentiality, and lack of trust in professionals [6, 7]. Young people experience the transition between mental health services (e.g. from child to adult mental health services) as poorly coordinated and dramatic[8, 9].

In Sweden, the prevalence of mental health problems among young people has increased dramatically by about 70% during the period 2006–2016, the highest increase among the Nordic countries. Depression, anxiety, and substance abuse have shown the largest increase [10, 11], and suicide rates have not decreased in younger age groups unlike in the overall population [12].

To enhance accessibility to mental health services for youth and facilitate coordination, the Swedish health system since 2009 has been developing a strategy to integrate youth mental health services within primary care services. The integration is labeled First Line Mental Health Services (FLMHS) [13, 14]. This approach has been used in several other countries [15, 16]. Research has shown that the integration of mental health services within primary care improves the management of common mental health problems among adults [16, 17] and children [18]. Youth between 15 and 25 years of age have found it easier to access mental health services when delivered through less stigmatized, more visible and familiar primary care services [19]. Reforms of youth mental health services have been implemented in many countries, but surprisingly, there is still limited research analyzing integrated care models such as the FLMHS [19-21].

The FLMHS was launched as a way to address three main challenges: (1) inequities in the mental health and social services provided to children and youth, (2) unclear division of responsibilities within and between health and social services, and (3) a need to develop

For peer review only

versatile health system solutions reflecting the diverse conditions prevailing in the different groups of the population and parts of the country. The establishment of FLMHS was in addition expected to relieve the high (unfulfilled) demand for specialized child mental health services and strengthen the collaboration between diverse public services and sectors [22, 23]. Currently, the sectors that are most frequently involved in FLMHS are primary health care centers, school health services and youth clinics (YC) [24]. More than 250 YCs have been in operation in Sweden since the 1970-1980s, providing youth-centered health services for young people. According to the Swedish Society for Youth Centers (FSUM) guidelines, the minimum staff of a YC includes a midwife and a social counsellor or psychologist, with a physician working part-time. However, many YCs (especially the ones located in bigger cities) also have nurses, dieticians, sexologists, and specialized medical doctors (e.g., psychiatrists, obstetricians). There can be cooperation with other services such as the school health services and child/adult psychiatry, or the local employment and health insurance offices. Young people contact the YC directly as outpatient visitors without referral, for cost-free consultation. Most YCs are placed off the premises of general health services, and in addition to consultations provided in the clinic they also work in promotion, mainly through school visits of 15-year-old pupils to the clinics [25, 26]. This national network of YCs offers an important opportunity to integrate mental health services within services that report a high level of trust by young people [27]. Youth commonly approach YCs with needs and questions that fall within the area of Sexual and Reproductive Health, e.g. requests for prescriptions for birth control pills, tests or treatment of sexually transmitted infections. The identity and role of YCs is well established among youth, YC health professionals and in other parts of the health system. However, ever since their establishment in the 1970s, YCs are also responding to youth needs in regard to mental health and mental health problems. YC have come to play a key role in the FLMHS where youth mental health services are integrated in primary care

For peer review only

level. YCs operate together with other stakeholders within the FLMHS and interact with stakeholders from the larger youth mental health system, e.g., authorities, specialized health services and schools (fig. 1).

<Figure 1 Stakeholders in and surrounding the FLMHS for youth >

For peer review only

In this paper, we present the protocol for a health policy and system study with an aim to analyze Swedish policy efforts to strengthen FLMHS for youth. We focus on YCs and adopt Atun's et al conceptual framework for investigating the integration of health interventions into health systems [28]. The research questions we aim to answer with this study are as follows: (1) How and under what circumstances are YCs part of the FLMHS for youth in the Swedish health system? (2) How can the integration of mental health services into YCs in the Swedish health system be strengthened?

Conceptual framework

The approach to mental health systems used in this study is inspired by complexity theory [29], with a specific focus on the integration of targeted health interventions within health systems [28]. Instead of portraying the different hierarchical levels and actors in the mental health system [22], complexity theory portrays them as interconnected and centered on the service user (fig. 2). In complex adaptive systems, individuals, services, and organizations are active agents that interact with each other, establishing dynamic interconnections [29].

<Figure 2 The Model for Mental Health Care Delivery as a Complex Adaptive System inspired by Ellis et al [29].>

For peer review only - http://bmjopen.bmj.com/site/about/guidelines.xhtml

This study will explore the intentions and strategies among national stakeholders and policy makers (e.g., authorities, politicians) in terms of integrating YCs in FLMHS and if and how YCs coordinate with other health actors in the FLMHS for youth, with specialized mental health services and with social services. The organization of this system is influenced by, and influences, both policies and research (Fig. 2).

Inspired by Atun et al. [28], we conceive integration as the process, pattern, and extent of adoption and eventual assimilation of new strategies into the health system (in this case, mental health services into YCs) that are embedded within FLMHS for youth.

In Figure 3, we have adapted Atun et al.'s framework to the specific intervention being analyzed in this study. Atun et al.'s conceptual framework provides a systematic approach to researching integration by focusing on 5 domains that influence the adoption and diffusion of health interventions.

<Figure 3 Conceptual Framework to Analyze Integration of Mental Health Care for Youth within Swedish Youth Clinics>

The first domain relates to the nature of the problem being addressed, i.e., the understanding regarding the urgency and severity of the problem influences the speed of adoption [28]. In this protocol, we conceptualize the problem as the weaknesses of the health system to adequately respond to the increasing mental health needs of young people.

The second domain refers to the characteristics of the intervention. Here, Atun et al. point out that interventions that for example are perceived as having a relative advantage, are less complex and risky and are more easily integrated. In this protocol, the intervention to address

For peer review only

For peer review only - http://bmjopen.bmj.com/site/about/guidelines.xhtml

this problem is the integration of mental health services within the existing network of Swedish YCs.

The third domain refers to the adoption system, namely the key actors and institutions involved in the integration [28]. Each stakeholder has differing perceptions of the benefits, risks, and legitimacy of the intervention, and the different interests at stake have to be explored. The adoption system in this study focuses on the YC care providers, the managers, and the young service users.

The fourth domain refers to the characteristics of the health system [28]. With regard to the characteristics of the health system, we are interested in the larger mental health system (i.e., national governance, primary and specialized mental health and social services), but with special focus on the sub-system of FLMHS for youth. The focus is on analyzing the collaboration between the services responsible for FLMHS for youth and how the different ways of organizing them and their approaches toward young people influence both the integration of mental health services within YCs and the collaborative dynamics with the other services.

The fifth domain refers to the broader socio-cultural context, where critical events, prevailing political economy, and socio-cultural norms also affect integration [28]. The broader context in the study protocol refers to how the current political economic situation and the socio-cultural characteristics of Swedish society, influence the integration of mental health services for youth within YCs.

For peer review only - http://bmjopen.bmj.com/site/about/guidelines.xhtml

Material and methods

Study design

To address our two main research questions, two methodological approaches will be combined in a sequential way: (1) a realist evaluation [30] and (2) concept mapping [31]. The study design will follow an iterative analytical process in which the conduct of the concept mapping will draw on the findings from the realist evaluation. Qualitative and quantitative data collection and analysis will be combined to best capture the complexity of the phenomenon under investigation. The integration of qualitative and quantitative approaches enables researchers to explore phenomena from different perspectives and provides an enriched understanding [32]. The data collection started in November 2019 and will be finalized in November 2022. The main research questions and the methodologies applied are summarized in Table 1.

Table 1: The research process

Research question in focus	Methodological approach	Steps	Methods for data collection	Methods for data analysis
How and under what circumstances are YCs part of the FLMHS for youth in the Swedish health care system?	Realist evaluation	Eliciting the program theory	Literature review<br>Documentary review<br>Interviews with stakeholders	Thematic analysis
		Testing the program theory in selected cases	Analytical multiple case study<br>Questionnaires for mental health care consultations	Descriptive statistical analysis
		Refining the program theory	Interviews with professionals from YCs	Thematic analysis
			Interviews with professionals from other services involved in FLMHS for youth<br>Interviews with professionals at specialized services<br>Photo-elicitation with young people	Thematic analysis
How can the integration of	Concept mapping	Presenting the findings and	Workshop and photo exhibition with (1) professionals working in	

For peer review only

For peer review only - http://bmjopen.bmj.com/site/about/guidelines.xhtml

mental health care services into YCs in the Swedish health system be strengthened?	engaging professionals and young people	YCs, (2) professionals in other FLMHS services for youth and in managerial positions, and (3) young people and youth organizations	
	Developing the concept maps	Brainstorming, pile sorting and rating, and map development using Concept System-CS Global Max	Concept mapping techniques (mixed methodology combining multivariate statistical methods, and collective qualitative interpretation)
	Bringing everything together and proposing actions	Final workshop	

For peer review only

Patient and public involvement

The research questions in this study have been informed by the experiences and preferences of youth, through our previous studies exploring youth views on YCs. Furthermore, the study adopts a participatory approach which has implications for the design, conduct of the study and also the choice of outcome measures.

All phases of the study will be presented and discussed with stakeholders at youth clinics (both managers and professionals) and other arenas (i.e. youth organizations) before and during data collection. Dissemination is a core component of the second phase. Stakeholders (including young people) will be invited to workshops to discuss preliminary results and engage in a concept mapping to collectively develop a set of strategies to improve the integration of mental health services within YCs. One of the main outcomes in this study is to reach consensus on the strategies to strengthen the integration of youth clinics in the FLMHS.

Study setting

The responsibility for providing health services in the Swedish health care system is shared between the national government, the regions, and the municipalities. The Swedish Health

For peer review only - http://bmjopen.bmj.com/site/about/guidelines.xhtml

Care Act gives a commitment to provide "good health and care on equal terms for the entire population" [33]. However, evaluations have shown that evidence-based health services are inaccessible to children and youth with mental health problems to an extent that diverges from the Health Care Act [34].

Data collection and analysis phase I - Formative realist evaluation

To answer the first research question, a formative realist evaluation of the integration of mental health services within YCs will be conducted. Realist evaluation is well suited to analysis of complex interventions aiming to ascertain whether the intervention works but also why, for whom, and under what circumstances [30]. In realist evaluation, theories are at the middle-range level, connecting the intervention with contextual factors, with the mechanisms that the intervention triggers within the target populations, and with the outcomes achieved. The aim is to identify patterns of intervention-context-mechanism-outcomes that can explain how, why, and under what circumstances a specific intervention works.

Pawson [30] describes cycles of realist evaluation, starting from eliciting the program theory behind the implementation of a particular intervention, followed by putting this theory under test in specific cases and coming up with a refined program theory. The entire process should be conducted in close dialogue with stakeholders involved in the development and implementation of the intervention, because the aim is to come up with a revised and improved intervention [30].

Realist evaluation can be applied in a summative way (once an intervention has ended or come to a conclusion) or in a formative way (while an intervention is being implemented). Integrated care programs for young people's mental health need to adapt and learn continuously, therefore we view our realist evaluation through the lens of formative evaluations, aiming for continuous quality improvement. The realist evaluation will cover the

For peer review only - http://bmjopen.bmj.com/site/about/guidelines.xhtml

following steps: (1) developing the program theory, (2) testing the program theory in selected cases, (3) refining the program theory.

Developing the program theory

The realist evaluation will start developing the program theory through a review of the literature and policy documents. The scientific literature, policies, and other relevant documents related to the intervention will be reviewed, looking for key concepts, ideas, and theories that can help to explain how the integration of mental health services for youth within the FLMHS is supposed to work. The scientific literature and documents will be analyzed using thematic analysis following Clarke & Braun [35].

In addition, we will interview national- and regional-level stakeholders involved in developing the intervention (i.e., the Swedish Association of Local Authorities and Regions, The Swedish Association of Youth Clinics). The interviews will aim to make the program theory behind the intervention explicit, namely the integration of mental health services within YCs, and to analyze how the increasing problems of youth mental health and the planned solutions/responses are understood. The five domains of Atun et al.'s framework [28] in relation to the three key concepts of realist evaluation (context, mechanisms, outcomes) will be the main topics explored. The interviews will be audio-recoded, transcribed, and analyzed using abductive thematic analysis [35], moving back and forward between our conceptual framework and the data. The program theory that develops will explain how the intervention is supposed to work. Following the ideas of FLMHS [22], the outcomes will mainly focus on whether the YCs (1) are accessible, (2) allow early detection, (3) have broad expertise of good quality, (4) have good reporting and monitoring systems, and (5) have good collaboration with other services [22].

For peer review only - http://bmjopen.bmj.com/site/about/guidelines.xhtml

Testing the program theory in selected cases

In the next phase, the program theory will be put to the test in 3–5 specific cases: YCs and the FLMHS in which they are embedded, including the surrounding referral services. To capture diverse contextual circumstances, we will choose clinics in different municipalities, located in different regions, and embedded in different models of FLMHS for youth (as defined by the Swedish Association of Local Authorities and Regions [22]).

From each case, quantitative information will be gathered on the extent and characteristics of mental health consultations. For this study, we will distribute the questionnaire to all the professionals working at the YC in the 3–5 cases, including midwives, psychologists, social workers, medical doctors, and other staff who are frequently approached by young people to discuss mental health issues. The questionnaire will be applied over 6 months in each of the clinics participating as cases. Analysis of the quantitative data will offer answers whether the services (1) are accessible, (2) allow early detection, and (3) offer good quality services [23].

Qualitative information from each case will include interviews (~5–10 per case) with (1) professionals working in the YC, (2) professionals working in other FLMHS for youth (e.g., student health, health centers, social services), and (3) professionals working in specialized mental health services. Because the way different municipalities are implementing FLMHS for youth and the services with responsibility for this sub-system vary, the institutions represented might vary from case to case. Professionals working in the YCs will include the head of the clinic and a variety of professionals, including professions more focused on mental health (e.g., counselor, psychologist) and others (e.g., midwives). Interviews at this stage will focus on health professionals' experiences of mental health consultations and if and how mental health services have been integrated and possibly sustained within the clinic's practice, coordination within the clinic and with other health care resources (both first-line and specialized) and social resources. Professionals working in FLMHS for youth will include

For peer review only - http://bmjopen.bmj.com/site/about/guidelines.xhtml

those working in, for example, primary health care centers and school health services. Specialist mental health professionals will include those working in child mental health services and adult mental health services. Interviews with staff in specialized mental health services will focus on how they perceive the role of YCs in FLMHS for youth, and their collaboration with YCs and with other services. Data from the interviews will be analyzed using thematic analysis [35].

To capture the views and experiences of young people (aged 16–29 years), we will engage young people in an auto-driven photo-elicitation interview to gather their perspectives [36]. Photo-elicitation is a research method that incorporates images in the interview process to elicit participants' subjective explanations. Photo-elicitation has gained recognition as a way to better capture young people's attention, ease rapport, and balance power differentials.

From each case, 6–8 youths aged 16–29 years will be recruited. Participants will include youth with experience using YC mental health services and those who do not have that experience. For the latter, we will recruit young people from high schools and universities. First, each participant will be approached and receive information about the study, offered a digital camera for 2 months and asked to take pictures to depict young people experiences and/or expectations of mental health and/or mental health services in the area where they live. At a second meeting, the participant will select 3 of the pictures taken as the basis for an interview, where topics around youth mental health, access to services, and experiences of them (with special focus on YCs) will be discussed.

All the interviews will be audio-recorded and transcribed. Detailed descriptions of the cases will be developed and further analyzed using an abductive thematic analysis approach, guided by the framework presented above (fig. 2). This analysis will inform the intervention implementation, contextual factors, potential mechanisms, and outcomes.

For peer review only - http://bmjopen.bmj.com/site/about/guidelines.xhtml

Refining the program theory

The initial program theory will be refined based on the findings in the theory testing to specify a middle-range theory that describes what works in terms of integrating mental health services for young people within YCs, for what type of youth subpopulations, and under what contextual circumstances [30, 37].

Data collection and analysis phase II – A concept mapping study

Building upon the knowledge gained during the previous phase, we will engage in a participatory process in which professionals and young people will be invited to take part in workshops followed by a concept mapping study [31]. The aim of this study is to develop strategies to strengthen the integration of mental health services within YCs. The concept mapping entails three steps: presenting findings and engaging professionals and young people in participatory workshops, developing the concept maps, and bringing everything together and proposing actions.

Presenting findings and engaging professionals and young people in participatory workshops

To present the preliminary results of the study, workshops will be arranged with 3 different types of stakeholders: (1) professionals working in YCs, (2) professionals in other FLMHS for youth and in managerial positions, and (3) young people and youth organizations. Youth will be recruited to the workshop using three strategies: first, the youth who were involved in the previous interview study in the project will be asked to participate in the workshop; second, YCs involved in the case studies will be asked to invite youth through advertising in waiting rooms/reception areas; and third, youth delegations will be asked to nominate youth to participate in the workshop.

For peer review only

Developing the concept maps

Using the findings from the participatory workshops as a basis for reflection, participants will be asked to participate in a concept mapping study to collectively develop a set of strategies to improve the integration of mental health services within YCs.

Concept mapping enables groups of actors to visualize their ideas about an issue of mutual interest and to develop common frameworks through a structured, participatory process. Qualitative and quantitative data are generated and integrated by participants through sequential steps, beginning with the generation of ideas (brainstorming), structuring of ideas through sorting and rating, development of conceptual maps based on multivariate statistical methods, and collective interpretation of the maps [31]. Data analysis will adopt concept mapping techniques with a mixed methodology combining multivariate statistical methods, and collective qualitative interpretation.

The research team will develop a focus question to guide the brainstorming activity and with clear instructions to all workshop participants and other relevant stakeholders and young people. Once the refined list of actions is developed, it will be sent again to the participants for sorting and rating. Three different sets of maps (one for each group) will be developed. The data will be analyzed using concept mapping techniques, which facilitate visualization of thematic clusters and identification of areas of consensus for action. The Concept System-CS Global Max software will be used throughout the process.

Bringing everything together and proposing actions

Finally, the concept maps and all the qualitative and quantitative results will be synthesized to answer our main research question: "How can the integration of mental health services into YCs in the Swedish health system be strengthened?" Building on the concept maps and

For peer review only - http://bmjopen.bmj.com/site/about/guidelines.xhtml

synthesized results, a final workshop will be held with young people and relevant stakeholders to discuss the maps and come up with a set of elaborated strategies.

Ethics and dissemination

This study has received approval from the Swedish Ethical Review Agency (Dnr 2019-2910, 2020-04720) and complies with the Helsinki Declaration. All participants will be informed that participation in the study is voluntary. Individuals will be provided with information in verbal and written format, and offered the opportunity to ask any questions. Written informed consent will be obtained from all participants.

Results will be published in open-access peer-reviewed journals and presented at scientific conferences, and as described before, preliminary findings will be discussed with stakeholders during the concept mapping.

Discussion

This paper presents a study protocol using a multiple case-study design to understand the integration of mental health services within YCs with a view to strengthening FLMHS for youth in Sweden and to develop strategies for strengthening this process.

This study is timely as it goes hand in hand in informing policy efforts to integrate mental health services within YCs and YCs within the FLMHS for youth in Sweden. Atun et al.'s [28] framework has been used to analyze the integration of programs on communicable diseases, cardiovascular disease, and sexual and reproductive health interventions, but, to our knowledge, it has never been used to analyze the integration of mental health services in

For peer review only - http://bmjopen.bmj.com/site/about/guidelines.xhtml

primary care. The methodological approach, involving realist evaluation and concept mapping, combines quantitative and qualitative methods sequentially in a way that fits the research questions and the health systems approach to complexity, and is innovative in the field of public health.

The study combines two methodological approaches: realist evaluation [30] and concept mapping [31]. Unlike randomized controlled trials, realist evaluation does not aim to control contextual factors but rather to explore how outcomes of an intervention interact with the context in which it is implemented. Realist evaluation is a methodology that rests on critical realism, and it mainly focuses on individual mechanisms in understanding an intervention. In this study, we do not focus only at the individual level but also the team and organizational levels. This challenges the method to some degree, but we have good experiences of using the method in this broader sense, for example, in the context of primary health care services [38].

Concept mapping is a mixed participatory method for involving stakeholders and youth in a structured conceptualization process. It has been developed to collect and integrate input from multiple sources and stakeholders with various demand, expertise or interest [39]. One challenge in using concept mapping is that the methodology utilizes group processes, for example, in brainstorming activities. Interaction within groups and the group processes are affected by entities such as power and status [40]. As participants are diverse in terms of age, education, and gender, we see a potential risk that participants will not have equal possibilities to contribute in the concept mapping. To balance participant contributions in group-oriented tasks, we will have this challenge in mind as we put the groups together. The moderator of the session will also be instructed to facilitate the participation of all group members. By taking these measures, we hope to balance the potential negative impact of the group processes to some degree.

For peer review only - http://bmjopen.bmj.com/site/about/guidelines.xhtml

The results of the study are expected to contribute new knowledge concerning the process of integration of YC in the FLMHS for youth but also to lead to recommendations for policy makers and decision makers within the field of youth mental health services.

Covid-19 pandemic: adjustments

Due to the Covid-19 pandemic some adjustments to the planned methodology have been, and will be necessary. During year 2020 and 2021 meetings and travels have been restricted in Sweden, with the purpose of minimizing spread of the Covid-19 infection. The restrictions have had implications for data collection, all interviews during the pandemic have been and will be performed digitally. The pandemic has also brought an increased strain on the health system in all levels, affecting the possibility to recruit participants operating within the health system, for example in government authorities or in youth clinics. This has and will be handled by adjusting the timeline forward and being flexible in terms of how interviews and workshops will be carried out (i.e. digital or face-to face).

Authors' contributions

IG designed the research proposal, drafted the application, and led the discussion in the group as principal investigator of the research proposal. LRS led the development of the first draft of the manuscript based on the research proposal and gathered comments from all team members to revise successive drafts and develop and submit the final draft of the manuscript to the journal. IG, MC, MW, and AKH are part of the research team that supported and developed the protocol. They contributed with ideas and discussed the protocol that was submitted and granted funding from FORTE. They read and critically commented on and revised successive drafts of the manuscript and approved the final version before submission to the journal.

For peer review only - http://bmjopen.bmj.com/site/about/guidelines.xhtml

Funding statement

This work was supported by the Swedish Research Council for Working Life and Living Conditions (FORTE) grant number 2018-00364.

Competing interests

The authors declare that they have no competing interests.

For peer review only

For peer review only - http://bmjopen.bmj.com/site/about/guidelines.xhtml

References

1. Bitsko RH, Holbrook JR, Ghandour RM et al (2018) Epidemiology and impact of health care provider-diagnosed anxiety and depression among US children. J Dev Behav Pediatr 39(5):395–403.

2. Pitchforth J, Fahy K, Ford T, Wolpert M, Viner RM, Hargreaves DS. Mental health and well-being trends among children and young people in the UK, 1995–2014: analysis of repeated cross-sectional national health surveys. Psychological Medicine. Cambridge University Press; 2019;49(8):1275–85.

3. Rickwood DJ, Deane FP, Wilson CJ. When and how do young people seek professional help for mental health problems? Med J Aust. 2007 Oct 1;187(S7):S35-9.

4. McGorry, PD; Mei, Cristina. Early intervention in youth mental health: progress and future directions. Evidence-based mental health, 2018, 21.4: 182-184.

5.Weinberger A, Gbedemah M, Martinez A, Nash D, Galea, S, Goodwin R. Trends in depression prevalence in the USA from 2005 to 2015: widening disparities in vulnerable groups. Psychol Med. 2018;48(8):1308–15.

6. Patel V, Flisher AJ, Hetrick S, McGorry P. Mental health of young people: a global public-health challenge. Lancet. 2007;369(9569):1302–13.

7. Malla A, Iyer S, McGorry P, Cannon M, Coughlan H, Singh S, et al. From early intervention in psychosis to youth mental health reform: a review of the evolution and transformation of mental health services for young people. Soc Psychiatry Psychiatr Epidemiol. 2016;51(3):319–26.

For peer review only

8. Gulliver A, Griffiths KM, Christensen H. Perceived barriers and facilitators to mental health help-seeking in young people: a systematic review. BMC Psychiatry 2010;10(1):113.

9. Broad KL, Sandhu VK, Sunderji N, Charach A. Youth experiences of transition from child mental health services to adult mental health services: a qualitative thematic synthesis. BMC Psychiatry. 2017;17(1):380–11.

10. Bremberg S. Mental health problems are rising more in Swedish adolescents than in other Nordic countries and the Netherlands. Acta Paediatr. 2015;104(10):997–1004.

11. Calling, S., Midlöv, P., Johansson, SE. et al. Longitudinal trends in self-reported anxiety. Effects of age and birth cohort during 25 years. BMC Psychiatry 2017;17, 119

12. National Board of Health and Welfare (Socialstyrelsen). Utvecklingen av psykisk ohälsa bland barn och unga vuxna till och med 2016. Stockholm: Socialstyrelsen; 2017.

13. Swedish Association of Local Authorities and Regions (Sveriges Kommuner och Regioner). Uppdrag Psykisk Hälsa. Första linjen för barn och ungas psykiska hälsa. Stockholm: SALAR; 2016.

14. Swedish Association of Local Authorities and Regions (Sveriges Kommuner och Regioner). Uppdrag Psykisk Hälsa. Första linje boken. Stockholm: SALAR; 2017.

15. World Health Organization. Integrating mental health into primary care: a global perspective. Geneva: World Health Organization; 2008.

16. Druss BG, Goldman HH. Integrating Health and Mental Health Services: A Past and Future History. American Journal of Psychiatry. 2018 Dec 1;175(12):1199–204.

For peer review only

For peer review only - http://bmjopen.bmj.com/site/about/guidelines.xhtml

17. Kates N, Arroll B, Currie E, Hanlon C, Gask L, Klasen H, et al. Improving collaboration between primary care and mental health services. The World Journal of Biological Psychiatry. 2019 Nov 26;20(10):748–65.

18. Campo JV, Geist R, Kolko DJ. Integration of pediatric behavioral health services in primary care: improving access and outcomes with collaborative care. Can J Psychiatry. 2018;63(7):432–38.

19. Hetrick SE, Bailey AP, Smith KE, Malla A, Mathias S, Singh SP, et al. Integrated (one-stop shop) youth health care: best available evidence and future directions. Med J Aust 2017;207(S10):S5–18.

20. Kinchin I, Tsey K, Heyeres M, et al. Systematic review of youth mental health service integration research. Aust J Prim Health. 2016;22(4):304–15.

21. Richardson LP, McCarty CA, Radovic A, Suleiman AB. Research in the integration of behavioral health for adolescents and young adults in primary care settings: a systematic review. J Adolescent Health. 2016;60(3):261–9.

22. Swedish Association of Local Authorities and Regions (Sveriges Kommuner och Regioner). Psykisk hälsa på ungdomsmottagningen – en kartläggning av Sveriges ungdomsmottagningar. Stockholm: SALAR; 2017.

23. National Board of Health and Welfare (Socialstyrelsen). Kartläggning av första linjens verksamhet för barn och unga med psykisk ohälsa. Stockholm: Socialstyrelsen; 2021.

24. Swedish Association of Local Authorities and Regions (Sveriges Kommuner och Regioner). Första linjen 2020 Nulägesbeskrivning av hälso- och sjukvårdens första linje för barn och ungas psykiska hälsa. Stockholm: SALAR; 2020

For peer review only

For peer review only - http://bmjopen.bmj.com/site/about/guidelines.xhtml

25. Strömbäck M, Malmgren-Olsson EB, Wiklund M. (2013) Girls need to strengthen each other as a group': experiences from a gender-sensitive stress management intervention by youth-friendly Swedish health services – a qualitative study. BMC Public Health 13, 907.

26. Thomée S, Malm D, Christianson M, Hurtig A.K, Wiklund M, Waenerlund, A.K, Goicolea I. (2016) Challenges and strategies for sustaining youth-friendly health services - a qualitative study from the perspective of professionals at youth clinics in northern Sweden. Reproductive health, 13(1), 1-13.

27. Goicolea I, Hultstrand Ahlin C, Waenerlund A.K, Marchal B, Christianson M, Wiklund M, et al. Accessibility and factors associated with utilization of mental health services in youth health centers. A qualitative comparative analysis in northern Sweden (Report). Int J Ment Health Syst. 2018;12(1):69.

28. Atun R, de Jongh T, Secci F, Ohiri K, Adeyi O. Integration of targeted health interventions into health systems: a conceptual framework for analysis. Health Policy Plan. 2009;25(2):104–11.

29. Ellis L, Churruca K, Braithwaite J. Mental health services conceptualised as complex adaptive systems: what can be learned? Int J Ment Health Syst 2017;11:43.

30. Pawson R. The science of evaluation: a realist manifesto. Thousand Oaks, CA: Sage; 2013.

31. Kane M, Trochim WM. Concept mapping for planning and evaluation. Thousand Oaks, CA: Sage; 2007

32. Palinkas L, Aarons G, Horwitz S, Chamberlain P, Hurlburt M, Landsverk J. Mixed method designs in implementation research. Adm Policy Ment Health. 2011;38(1);44–53.

For peer review only

For peer review only - http://bmjopen.bmj.com/site/about/guidelines.xhtml

33. The health care act. Minstry of health. Hälso- och sjukvårdslagen. Svensk författningssamling 1982:76. Stockholm: Socialdepartementet.

34. National Board of Health and Welfare. Socialstyrelsen. (2009) Barn- och ungdomspsykiatri – vård på olika villkor. Rapport från en nationell tillsyn. Stockholm.

35. Clarke V, Braun V. Thematic analysis. The journal of positive psychology. 2016;12(3):297–8.

36. Clark CD. The autodriven interview: a photographic viewfinder into children's experience. Vis Sociol. 1999;14(1):39–50.

37. Marchal B, van Belle S, van Olmen J. Is realist evaluation keeping its promise? A review of published empirical studies in the field of health systems research. Evaluation. 2012;18(2):192–212.

38. Goicolea I, Hurtig A-K, San Sebastian M, Marchal B, Vives-Cases C. Using realist evaluation to assess primary healthcare teams' responses to intimate partner violence in Spain. Gaceta Sanitaria. 2015;29(6):431–6.

39. Trochim, W., & Kane, M. (2005). Concept mapping: an introduction to structured conceptualization in health care. International Journal for Quality in Health Care, 17(3), 187–191.

40. Brown, R., & Pehrson, S. (2019). Group Processes: Dynamics Within and Between Groups. John Wiley & Sons, Incorporated.

For peer review only - http://bmjopen.bmj.com/site/about/guidelines.xhtml

Figure 1 Stakeholders in and surrounding the FLMHS for youth

Figure 2 The Model for Mental Health Care Delivery as a Complex Adaptive System inspired by Ellis et al [22]

Figure 3 Conceptual Framework to Analyze Integration of Mental Health Care for Youth within Swedish Youth Clinics

For peer review only

Figure 1.

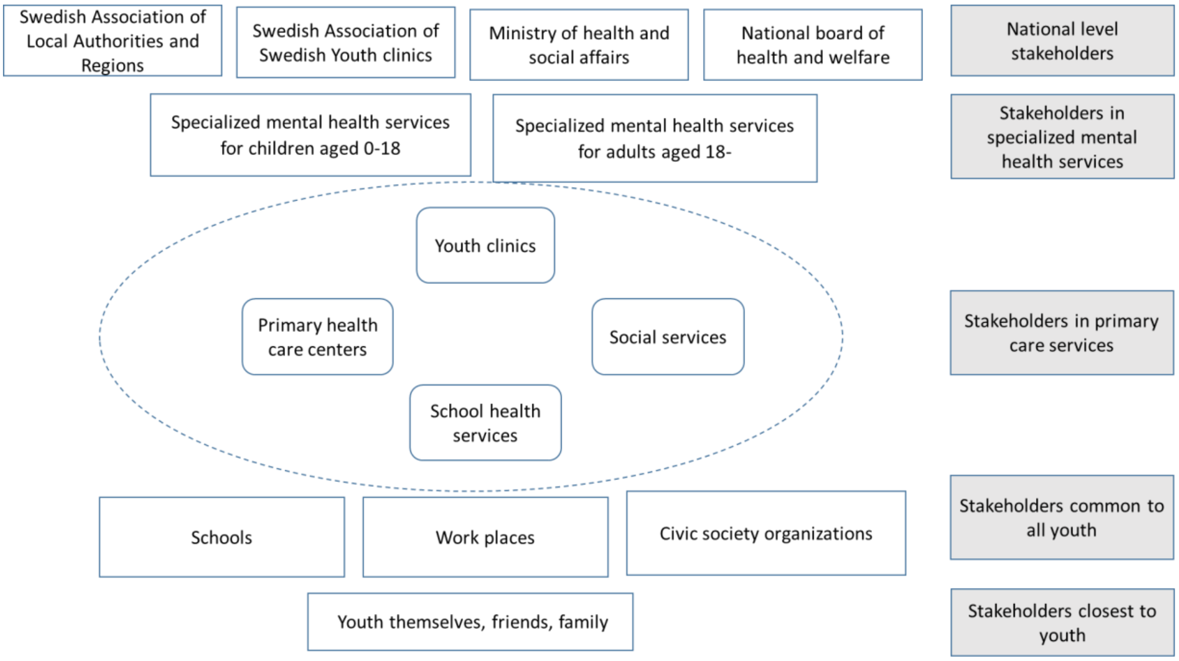

review only

Figure 2.

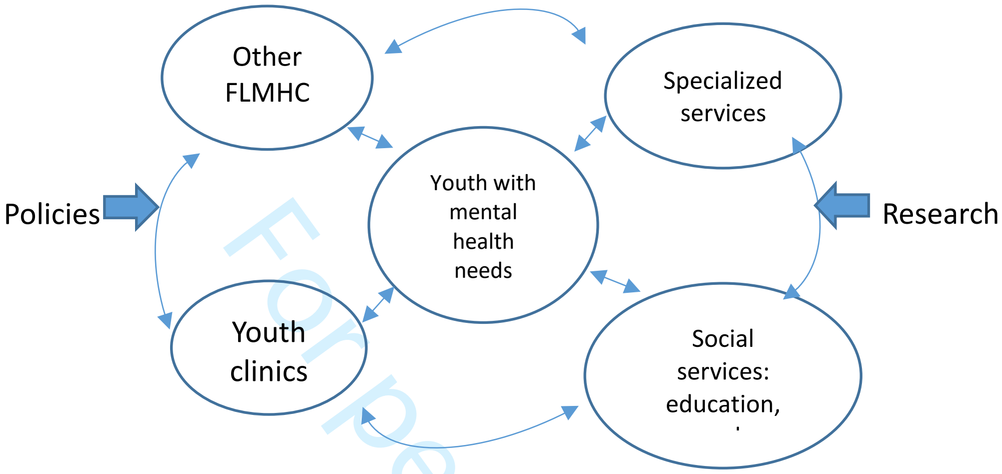

For peer review only

Figure 3

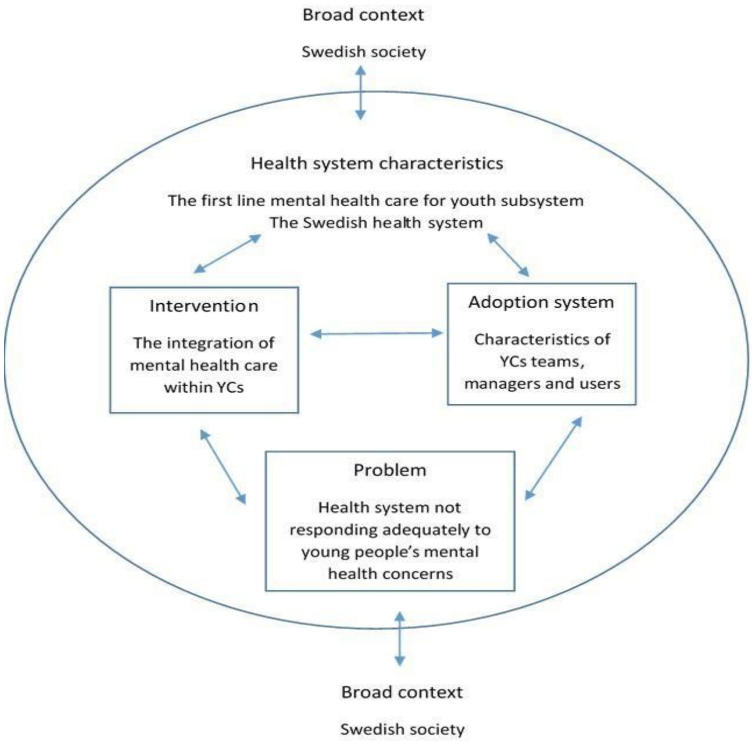

review only
